# Analogies of the classical Euler top with a rotor to spin squeezing and quantum phase transitions in a generalized Lipkin-Meshkov-Glick model

**DOI:** 10.1038/s41598-018-20486-y

**Published:** 2018-01-31

**Authors:** Tomáš Opatrný, Lukáš Richterek, Martin Opatrný

**Affiliations:** 1 Faculty of Science, Palacký University, 17. Listopadu 12, 77146 Olomouc, Czech Republic; 2 Faculty of Mechanical and Industrial Engineering, Vienna University of Technology, Getreidemarkt 9, 1060 Wien, Austria

## Abstract

We show that the classical model of Euler top (freely rotating, generally asymmetric rigid body), possibly supplemented with a rotor, corresponds to a generalized Lipkin-Meshkov-Glick (LMG) model describing phenomena of various branches of quantum physics. Classical effects such as free precession of a symmetric top, Feynman’s wobbling plate, tennis-racket instability and the Dzhanibekov effect, attitude control of satellites by momentum wheels, or twisting somersault dynamics, have their counterparts in quantum effects that include spin squeezing by one-axis twisting and two-axis countertwisting, transitions between the Josephson and Rabi regimes of a Bose-Einstein condensate in a double-well potential, and other quantum critical phenomena. The parallels enable us to expand the range of explored quantum phase transitions in the generalized LMG model, as well as to present a classical analogy of the recently proposed LMG Floquet time crystal.

## Introduction

“*The same equations have the same solutions*” is a well known Feynman’s quote from his lecture on electrostatic analogs^1^. Taking advantage of known solutions of Maxwell’s equations, Feynman shows how to apply them for solving problems of heat transport, neutron diffusion, fluid dynamics, and photometry. The message is that analogs are powerful tools that allow the exchange of know-how between different branches of physics. Here we follow this approach and focus on quantum analogs of the Euler dynamical equations, initially introduced to study rotations of rigid bodies. We show that already the simplest version of Euler equations describing a free spinning top is relevant to the quantum mechanical problem of spin squeezing^2,3^, i.e., noise suppression important for improving precision of atomic clocks and measuring devices^4–6^. These effects have their classical counterparts in dynamics of wobbling plates^7–9^, tennis racket instability^10,11^ or the Dzhanibekov effect^12^. If a freely spinning rotor with its axis fixed with respect to the top is added, plethora of new phenomena occur with analogies across diverse fields. In the quantum case one can observe features of the Lipkin-Meshkov-Glick (LMG) model of nuclear physics^13^ with various critical phenomena^14^ including quantum phase transitions with their generalization to excited state quantum phase transitions^15,16^, self-trapping of Bose-Einstein condensates in potential wells^17,18^, or twist-and-turn scenario of spin squeezing^19^. These quantum phenomena correspond to purely classical effects such as satellite stabilization by momentum wheels^20,21^ or motion of an athlete executing a twisted somersault^22,23^.

Taking advantage of these analogies, we introduce new types of excited state quantum phase transitions in a generalized LMG model that correspond to different kinds of motion in rigid body dynamics. As another application we propose a classical version of the recently introduced LMG Floquet time crystal^24^.

## General features of the dynamics

### Classical model

**Figure 1:**
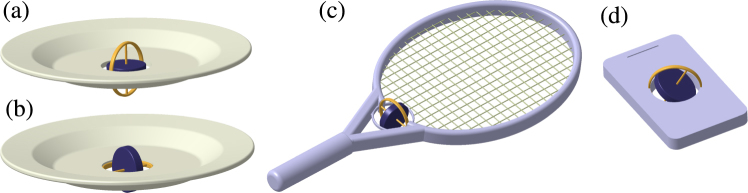
Examples of rigid bodies with a rotor. (**a**) Symmetric top, coaxial rotor; (**b**) symmetric top, perpendicular rotor; (**c**) asymmetric top, rotor with axis along one of the principal axes; (**d**) asymmetric top, general orientation of the rotor.

Evolution of the angular momentum $\overrightarrow{L}$ of a rigid body is governed by the equation $d\overrightarrow{L}/dt=\overrightarrow{M}$, where $\overrightarrow{M}$ is the torque. Assume that the torque stems from a rotor whose axis is fixed with respect to the rigid body as in Fig. 1. In the rotating coordinate system with axes fixed along the principal axes of the rigid body the evolution of the angular momentum vector $\overrightarrow{\omega }$ is given by

1 $${\dot{\omega }}_{j}=\frac{{I}_{k}-{I}_{l}}{{I}_{j}}{\omega }_{k}{\omega }_{l}+\frac{{K}_{k}{\omega }_{l}-{K}_{l}{\omega }_{k}}{{I}_{j}},$$

where the indexes *j*, *k*, *l* form an even permutation of 1, 2, 3, *L*_*k*_ = *I*_*k*_*ω*_*k*_, and *I*_1,2,3_ are the principal moments of inertia (see the Supplementary material for detailed derivation). These are the well known Euler dynamical equations which for $\overrightarrow{K}=0$ correspond to a free top, and here the special case corresponds to the torque coming from the rotor. It is suitable to work with the total angular momentum $\overrightarrow{J}\equiv \overrightarrow{L}+\overrightarrow{K}$, for which one finds

2 $${\dot{J}}_{j}=(\frac{1}{{I}_{l}}-\frac{1}{{I}_{k}}){J}_{k}{J}_{l}+\frac{{K}_{k}}{{I}_{k}}{J}_{l}-\frac{{K}_{l}}{{I}_{l}}{J}_{k}\mathrm{.}$$

As can be checked, the evolution equations conserve the kinetic energy of the rigid body and the magnitude of the total angular momentum, i.e., ${\dot{E}}_{{\rm{body}}}=0$ and ${\dot{J}}^{2}=0$, where

3 $${E}_{{\rm{body}}}=\frac{{L}_{1}^{2}}{2{I}_{1}}+\frac{{L}_{2}^{2}}{2{I}_{2}}+\frac{{L}_{3}^{2}}{2{I}_{3}},\quad {J}^{2}={J}_{1}^{2}+{J}_{2}^{2}+{J}_{3}^{2}\mathrm{.}$$

Thus, the trajectories in the angular momentum space are intersections of the energy ellipsoid *E*_body_ = const and the total angular momentum sphere *J* = const, their centers being displaced by $\overrightarrow{K}$. This geometric interpretation is especially helpful for finding stationary angular momenta and determining their stability.

### Quantum model

Assume two bosonic modes described by annihilation operators $\hat{a}$ and $\hat{b}$ with total number of particles *N*. These operators commute as $[\hat{a},{\hat{a}}^{\dagger }]=[\hat{b},{\hat{b}}^{\dagger }]=1$ and the remaining commutators vanish. One can introduce operator $\hat{\overrightarrow{J}}$ with components defined as ${\hat{J}}_{x}=\frac{1}{2}({\hat{a}}^{\dagger }\hat{b}+\hat{a}{\hat{b}}^{\dagger })$, ${\hat{J}}_{y}=\frac{1}{2i}({\hat{a}}^{\dagger }\hat{b}-\hat{a}{\hat{b}}^{\dagger })$, and ${\hat{J}}_{z}=\frac{1}{2}({\hat{a}}^{\dagger }\hat{a}-{\hat{b}}^{\dagger }\hat{b})$, with $N={\hat{a}}^{\dagger }\hat{a}+{\hat{b}}^{\dagger }\hat{b}$. These operators satisfy the angular momentum commutation relations $[{\hat{J}}_{x},{\hat{J}}_{y}]=i{\hat{J}}_{z}$, $[{\hat{J}}_{y},{\hat{J}}_{z}]=i{\hat{J}}_{x}$, and $[{\hat{J}}_{z},{\hat{J}}_{x}]=i{\hat{J}}_{y}$. Assume a general quadratic Hamiltonian in the form $\hat{H}={\sum }_{k,l}\,{\chi }_{kl}{\hat{J}}_{k}{\hat{J}}_{l}+{\sum }_{k}\,{{\rm{\Omega }}}_{k}{\hat{J}}_{k}$, where the indexes *k*, *l* run through *x*, *y*, *z*, and we put *ħ* = 1. As shown in ref.^25^, the quantities *χ*_*kl*_ = *χ*_*lk*_ can be treated as components of a twisting tensor *χ*. By a suitable rotation of the coordinate system, the twisting tensor can be cast into diagonal form such that the Hamiltonian is

4 $$\hat{H}=\sum _{k=1}^{3}\,({\chi }_{k}{\hat{J}}_{k}^{2}+{{\rm{\Omega }}}_{k}{\hat{J}}_{k}),$$

where *χ*_*k*_ are the eigenvalues of the twisting tensor. The Heisenberg evolution equations $id\hat{A}/dt=[\hat{A},\hat{H}]$ then yield

5 $$\frac{d{\hat{J}}_{j}}{dt}=({\chi }_{k}-{\chi }_{l})\,({\hat{J}}_{k}{\hat{J}}_{l}+{\hat{J}}_{l}{\hat{J}}_{k})+{{\rm{\Omega }}}_{k}{\hat{J}}_{l}-{{\rm{\Omega }}}_{l}{\hat{J}}_{k},$$

Note that Hamiltonian (4) commutes with ${\hat{J}}^{2}$ which is a conserved quantity, ${\hat{J}}^{2}\equiv {\hat{J}}_{1}^{2}+{\hat{J}}_{2}^{2}+{\hat{J}}_{3}^{2}=\frac{N}{2}(\frac{N}{2}+1)$.

### Correspondence of the models

Equations (2) and (5) have the same structure, except for (2) being classical equations whereas (5) are operator equations with symmetrized products of operators. Thus, both models yield analogous predictions. The two sets of equations correspond to each other provided we make the change

6 $${\chi }_{k}\leftrightarrow -\frac{1}{2{I}_{k}},\quad {{\rm{\Omega }}}_{k}\leftrightarrow \frac{{K}_{k}}{{I}_{k}},\quad {\rm{or}}\quad {I}_{k}\leftrightarrow -\frac{1}{2{\chi }_{k}},\quad {K}_{k}\leftrightarrow -\frac{{{\rm{\Omega }}}_{k}}{2{\chi }_{k}}.$$

Note that the dimension of the quantities is set by our choice *ħ* = 1; to have the usual dimensionality, the relation between *χ*_*k*_ and *I*_*k*_ would be changed to ${\chi }_{k}\leftrightarrow -\hslash /\mathrm{(2}{I}_{k})$.

Since in Eq. (5) only the differences between the twisting tensor eigenvalues occur, the dynamics are not changed if a constant is added to all eigenvalues of *χ*, i.e., *χ*_*k*_ → *χ*_*k*_ + *χ*_0_. This transformation just shifts the Hamiltonian by a constant ${\chi }_{0}\,{\hat{J}}^{2}$. The same holds in the classical dynamics if the moments of inertia and the angular momentum of the rotor are modified as

7 $$\frac{1}{{I}_{k}}\to \frac{1}{{I}_{k}}+\frac{1}{{I}_{0}},\quad {K}_{k}\to \frac{{K}_{k}}{1+\tfrac{{I}_{k}}{{I}_{0}}}$$

with *I*_0_ independent of *k*. As a consequence, for any quantum system described by twisting tensor *χ* and frequency vector $\overrightarrow{{\rm{\Omega }}}$, one can find a classical rigid body characterized by tensor of inertia *I* supplemented with a rotor with angular momentum $\overrightarrow{K}$ such that the combined system has the same dynamics.

### Lipkin-Meshkov-Glick model

In 1965 Lipkin, Meshkov and Glick formulated a toy model of multiparticle interaction that can be, under certain conditions, solved exactly, and thus serve as a basis for testing various approximation methods^13^. Although the original motivation was in modeling energy spectra of atomic nuclei, the scheme turned out to be useful for studying interesting phenomena in more general systems. The LMG Hamiltonian has the form $\hat{H}=\varepsilon {\hat{J}}_{3}+V({\hat{J}}_{1}^{2}-{\hat{J}}_{2}^{2})+W({\hat{J}}_{1}^{2}+{\hat{J}}_{2}^{2})$, where *ε*, *V* and *W* are real parameters. The quadratic part of the Hamiltonian corresponds to the diagonal twisting tensor *χ* with *χ*_1_ = *W* + *V*, *χ*_2_ = *W* − *V* and *χ*_3_ = 0. Since any multiple of a unit tensor can be added to *χ* without changing the dynamics, any diagonal *χ* can be expressed in a form corresponding to the quadratic part of the LMG Hamiltonian. In particular, for a diagonal *χ* with terms *χ*_1_,*χ*_2_,*χ*_3_, by subtracting *χ*_3_ from all the diagonal terms, one gets the LMG parameters *W* = (*χ*_1_ + *χ*_2_)/2 − *χ*_3_ and *V* = (*χ*_1_ − *χ*_2_)/2. Since for general quadratic Hamiltonians the labeling of principal axes 1, 2, 3 is arbitrary, any quadratic Hamiltonian with the linear part parallel to one of the principal axes is equivalent to the LMG Hamiltonian. Thus, in the classical analogy, the LMG model corresponds to a freely rotating rigid body supplemented with a rotor with its rotational axis fixed along one of the principal axes of the body as in Fig. 1(a–c). The special case of *V* = 0 corresponds to a symmetrical top with a coaxial rotor as in Fig. 1(a), whereas for *V* = *W* the LMG model corresponds to a symmetric top with a perpendicular rotor as in Fig. 1(b).

## Free symmetric top, Feynman’s plate, and spin squeezing by one-axis twisting

As the simplest model, consider a symmetric top with *I*_1_ = *I*_2_ ≠ *I*_3_ with no rotor, i.e., *K*_*k*_ = 0. Equation (1) then simplify to

8 $${\dot{\omega }}_{1}=-\tilde{{\rm{\Omega }}}{\omega }_{2},\quad {\dot{\omega }}_{2}=\tilde{{\rm{\Omega }}}{\omega }_{1},\quad {\dot{\omega }}_{3}=0,$$

where

9 $$\tilde{{\rm{\Omega }}}\equiv \frac{{I}_{3}-{I}_{1}}{{I}_{1}}{\omega }_{3}=(\frac{1}{{I}_{1}}-\frac{1}{{I}_{3}}){J}_{3}\mathrm{.}$$

Solutions of Eq. (8) describe regular precession of the top as ${\omega }_{1}=A\,\cos \,\tilde{{\rm{\Omega }}}t$, ${\omega }_{2}=A\,\sin \,\tilde{{\rm{\Omega }}}t$, where the amplitude is $A=\sqrt{{\omega }_{1}^{2}+{\omega }_{2}^{2}}$. Thus, in the frame fixed with the body, the axis of rotation circles with frequency $\tilde{{\rm{\Omega }}}$ around the symmetry axis of the top. For $A\ll {\omega }_{3}$, i.e., for small angles between the rotation axis and the symmetry axis, an external observer sees the top wobbling with frequency ${\omega }_{3}+\tilde{{\rm{\Omega }}}$. Two extreme cases of the mass distribution in the top correspond to a flat, plate-like top with *I*_3_ → 2*I*_1_, and a rod-like top with *I*_3_ → 0. The plate-like top has $\tilde{{\rm{\Omega }}}\to {\omega }_{3}$ so that the wobbling frequency is ≈2*ω*_3_, and the rod-like top has $\tilde{{\rm{\Omega }}}\to -{\omega }_{3}$ so that the wobbling frequency tends to zero (one can see that when throwing up a spinning pencil).

Feynman in his book “Surely, You Are Joking, Mr. Feynman!”^7^ tells a story: “ *I was in the [Cornell] cafeteria and some guy*, *fooling around*, *throws a plate in the air*. *As the plate went up in the air I saw it wobble*, *and I noticed the red medallion of Cornell on the plate going around*. *It was pretty obvious to me that the medallion went around faster than the wobbling*. *I had nothing to do*, *so I start to figure out the motion of the rotating plate*. *I discover that when the angle is very slight*, *the medallion rotates twice as fast as the wobble rate*–*two to one*. *It came out of a complicated equation*!” Feynman was surely joking when telling this story to R. Leighton who collected Feynman’s memories, because the situation is just opposite: the wobbling is twice as fast as the rotation. This follows from the above arguments, and was clearly explained in notes^8,9^ published after Feynman’s book.

In quantum domain, Hamiltonian (4) reduces to

10 $${\hat{H}}_{{\rm{OAT}}}=\chi {\hat{J}}_{3}^{2}$$

with $\chi \leftrightarrow 1/(2{I}_{1})-1/(2{I}_{3})$. This Hamiltonian corresponds to the one-axis twisting (OAT) scenario of spin squeezing first proposed theoretically by Kitagawa and Ueda^2^: the Bloch sphere is twisted around axis *J*_3_. For states near the equator of the Bloch sphere with *J*_3_ ≈ 0, Hamiltonian (10) squeezes the uncertainty area such that noise in some quantum variable decreases and increases in another.

A careful observer could see the OAT squeezing in the classical motion, as well. When the top is spun around an axis lying in the symmetry plane, *ω*_3_ = 0, then $\tilde{{\rm{\Omega }}}=0$ and the rotational axis keeps its orientation. If the rotational axis is oriented slightly off the symmetry plane, it slowly precesses with a speed proportional to its deviation of the plane. If the initial orientations are randomly scattered around an axis in the plane of the top, after some time, the directions of the rotation become squeezed from one side and stretched perpendicularly.

## Free asymmetric top, tennis-racket instability, and two-axis countertwisting

Assume a rigid body with the principal moments of inertia *I*_1_ < *I*_3_ < *I*_2_. The classical motion is described by Eqs (1) and (2) with *K*_*k*_ = 0. As is well known, rotations around the two extreme principal axes 1 and 2 are stable, whereas rotation around the intermediate principal axis 3 is unstable. The dynamics was studied in detail in^10–12^. One can observe this behavior when throwing up a spinning tennis racket: the rotations are stable if the axis of rotation is along the handle (smallest moment of inertia) or perpendicular to the plane of the head of the racket (biggest moment of inertia), and unstable if the axis of rotation is in the plane of the head of the racket, perpendicular to the handle (intermediate moment of inertia). If the initial angular velocity direction is slightly off the stable axis, the rotation axis precesses around it, but if it is slightly off the unstable axis, it diverges away. Typically on Earth, one cannot observe the free spinning body for a long period. However, the effect is spectacular in zero gravity conditions, provided that the initial angular velocity direction is very close to the unstable axis. As the result, one can see the “Dzhanibekov effect” named after Russian cosmonaut Vladimir Dzhanibekov who observed it while in space in 1985^12^.

In quantum domain the corresponding Hamiltonian can be cast into the form

11 $$\hat{H}={\chi }_{+}{\hat{J}}_{2}^{2}-{\chi }_{-}{\hat{J}}_{1}^{2}$$

with *χ*_+_ = 1/(2*I*_3_ )− 1/(2*I*_2_) and *χ*_−_ = 1/(2*I*_1_) − 1/(2*I*_3_). In the special case of *I*_3_ = 2*I*_1_*I*_2_/(*I*_1_ + *I*_2_) the Hamiltonian of (11) takes the form

12 $${\hat{H}}_{{\rm{TACT}}}=\chi ({\hat{J}}_{2}^{2}-{\hat{J}}_{1}^{2})$$

with *χ* = (*I*_2_ − *I*_1_)/(4*I*_1_*I*_2_). Hamiltonian (12) corresponds to the two-axis countertwisting (TACT) scenario of spin squeezing^2^: the Bloch sphere is twisted in one sense around *J*_1_ and in the opposite sense around *J*_2_. Spin states initially polarized along *J*_3_ become squeezed as the uncertainty circle is stretched in one direction and compressed in the other.

## Symmetric top with a coaxial rotor, spin twisting with coaxial rotation

Adding a rotor to the top or a linear term to the Hamiltonian makes the dynamics richer. Consider Hamiltonian in the form

13 $$\hat{H}=\chi {\hat{J}}_{3}^{2}+{\rm{\Omega }}{\hat{J}}_{3}$$

which corresponds to the LMG with *V* = 0, *ε* = Ω and *W* = −*χ*. Since the Hamiltonian is a function of ${\hat{J}}_{3}$, its eigenfunctions are Dicke states with sharp values of *J*_3_. The dynamics are split into two possible phases: dominant rotation with 2*J*|*χ*| < |Ω|, and dominant nonlinearity with 2*J*|*χ*| > |Ω|. In the case of dominant rotation the eigenstates corresponding to the extreme eigenvalues of $\hat{H}$ coincide with the eigenstates corresponding to the extreme eigenvalues of ${\hat{J}}_{3}$. In case of dominant nonlinearity either the ground or the highest excited state of $\hat{H}$ is one of the intermediate Dicke states.

In classical dynamics the situation corresponds to a symmetric top, *I*_1_ = *I*_2_ ≠ *I*_3_, with a coaxial rotor, *K*_1_ = *K*_2_ = 0 ≠ *K*_3_ ≡ *K*, as in Fig. 1(a). The equations of motion and their solution have the same form as those of a free symmetric top, Eq. (8), but the precession frequency is changed to

14 $$\tilde{{\rm{\Omega }}}=\frac{({I}_{3}-{I}_{1}){\omega }_{3}+K}{{I}_{1}}=(\frac{1}{{I}_{1}}-\frac{1}{{I}_{3}}){J}_{3}+\frac{K}{{I}_{3}}.$$

Similarly to the quantum case, the dynamics are split into two regimes with dominant rotation |*K*|/*J* > |1 − *I*_3_/*I*_1_|, and dominant nonlinearity |*K*|/*J* < |1 − *I*_3_/*I*_1_|. As an example, consider a plate with *I*_1_ = *I*_2_ = *I*_3_/2. In this case Eq. (14) yields $\tilde{{\rm{\Omega }}}={\omega }_{3}+2K/{I}_{3}$. Choosing $K=-\frac{1}{2}{I}_{3}{\omega }_{3}$ leads to $\tilde{{\rm{\Omega }}}=0$ so that the wobble frequency is equal to the rotation frequency, ${\omega }_{3}+\tilde{{\rm{\Omega }}}={\omega }_{3}$. As another example choose $K=-\frac{3}{4}{I}_{3}{\omega }_{3}$. This leads to $\tilde{{\rm{\Omega }}}=-{\omega }_{3}/2$ so that the wobble frequency of a plate is half the rotation frequency, ${\omega }_{3}+\tilde{{\rm{\Omega }}}={\omega }_{3}/2$. Thus, with a little cheating of adding a properly spinning rotor, one can force a plate to behave exactly as described in Feynman’s cafeteria story^7^.

## Symmetric top with a perpendicular axis rotor, twist-and-turn Hamiltonian

Let the Hamiltonian have the form

15 $$\hat{H}=\chi {\hat{J}}_{1}^{2}+{\rm{\Omega }}{\hat{J}}_{3}\mathrm{.}$$

**Figure 2:**
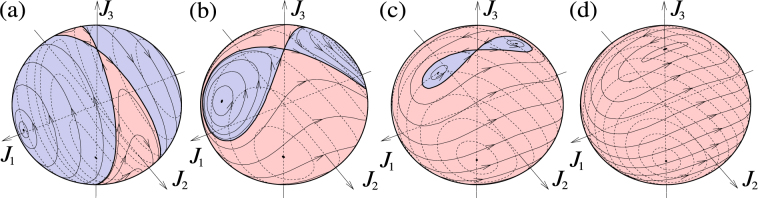
Angular momentum trajectories for a symmetric top with a perpendicular axis rotor, or a twist-and-turn Hamiltonian (15). The parameters are Ω/(*χJ*) = 0.2 (**a**), 1 (**b**), 1.7 (**c**), and 2 (**d**). Panels (a–c) correspond to the Josephson regime with the blue area representing “self-trapped” states. Panel (d) corresponds to the boundary between the Josephson and Rabi regimes where one unstable and two stable stationary points merge, leaving behind one stable stationary point for Ω/(*χJ*) > 2.

The corresponding evolution is twisting around axis *J*_1_ and simultaneous rotation about the perpendicular axis *J*_3_ (also called “twist-and-turn” dynamics^19^). As in the preceding case there are two distinct regimes: that with dominant rotation |Ω| > 2*J*|*χ*| called “Rabi regime”, and that with dominant twisting |Ω| < 2*J*|*χ*| called “Josephson regime” (see Fig. 2). In the Rabi regime, the Hamiltonian is nondegenerate, with a single maximum and a single minimum on the Bloch sphere. For 2*Jχ* = ±Ω a quantum phase transition occurs with the energy maximum or minimum being split into two, so that in the Josephson regime a saddle point on the Bloch sphere occurs. Physically the dynamices corresponds, e.g., to coherent atomic tunneling between two zero-temperature Bose-Einstein condensates confined in a double-well trap^17^, or to evolution of a two-component condensate^18^. In the Josephson regime, the condensate can be “self-trapped” in one of the wells^17^.

In classical dynamics the model corresponds to a symmetrical top with a perpendicular rotor such as in Fig. 1(b). The “Rabi oscillations” occur in the case with dominant rotor angular momentum, |*K*_3_|/*J* > |1 − *I*_3_/*I*_1_|: the rotational axis of the top circles around the axis of the rotor. If the axis of the body rotation is along the rotor axis, its direction is fixed and stable for both co-rotational and counter-rotational orientations. In the “self trapping” or “Josephson” regime, the angular momentum of the top is dominant, |*K*_3_|/*J* < |1 − *I*_3_/*I*_1_|. In this case, the counter-rotation becomes unstable: the direction opposite to the rotation of the rotor becomes located on a separatrix dividing the 4*π* sphere of rotational axis orientations into three regions (see Fig. 2(a–c) for visualization). In one region the rotational axis circles around the direction of the rotor, in the two other regions the rotational axis circles around a direction pointing between the rotor axis and the symmetry axis of the top.

## Excited quantum phase transitions in generalized LMG

**Figure 3:**
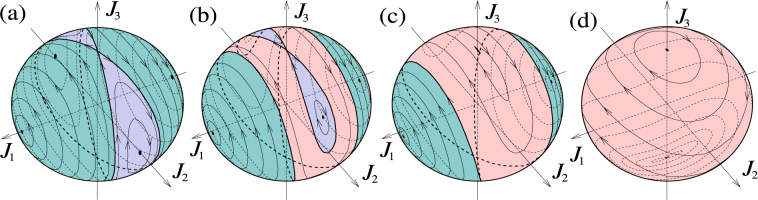
Angular momentum trajectories for an asymmetric top with a rotor along the middle axis. The twisting parameters of the corresponding quantum Hamiltonian are *χ*_3_ = 0, and *χ*_1_ = −10*χ*_2_. (**a**) Ω_3_ = 0 (i.e., no rotor), (**b**) |Ω_3_| = 1.7*J*|*χ*_2_|, (**c**) |Ω_3_| = 2*J*|*χ*_2_| (critical value for disappearing the saddle point along +*J*_3_), and (**d**) |Ω_3_| = 2*J*|*χ*_1_| (critical value for disappearing the saddle point along −*J*_3_).

Features of the LMG Hamiltonian have been widely explored, especially with focus on quantum phase transitions and related critical phenomena^26–32^. The concept of quantum phase transition typically refers to closing the gap between the ground and the first excited state by varying a system parameter. Recently the concept has been generalized to excited state quantum phase transitions (ESQPT)^15,16^, where the variation of parameters leads to sudden emergence of singularities in the energy spectrum. These effects can be related to the Hamiltonian map on the Bloch sphere (see Figs 2 and 3): a discontinuity in the energy spectrum corresponds to a local minimum or maximum of energy on the sphere, and a peak in the energy spectrum corresponds to a saddle point of energy.

### Stationary angular momenta and their stability

Even though the possibility to generalize LMG to arbitrary directions of the linear term was briefly mentioned in^33^, we are not aware of any systematic study of such a model. Here we classify phases in the generalized LMG given by Hamiltonian (4) by identifying the stationary angular momenta and finding their stability in the limit *N* → ∞ (so called thermodynamic limit). In the angular momentum space, stationary values correspond to the points where the constant energy ellipsoid touches the constant total-angular-momentum sphere. This occurs where the gradient of energy and the gradient of the squared total momentum of Eq. (3) are colinear, grad *E*_body_ = *λ* grad *J*^2^ for some *λ*. Eliminating *λ* and expressing in the vector equation the components *J*_1,2_ by means of *J*_3_ as *J*_1_ = *I*_3_*K*_1_*J*_3_/[(*I*_3_ − *I*_1_)*J*_3_ + *I*_1_*K*_3_], *J*_2_ = *I*_3_*K*_2_*J*_3_/[(*I*_3_ − *I*_2_)*J*_3_ + *I*_2_*K*_3_], which, when used in Eq. (3), leads to the polynomial equation for *J*_3_,

16 $$\sum _{n=0}^{6}\,{a}_{n}{J}_{3}^{n}=\mathrm{0,}$$

where the coefficients *a*_*n*_ are functions of *I*_1,2,3_ and *K*_1,2,3_ (or, equivalently, *χ*_1,2,3_ and Ω_1,2,3_) and *J*, and are expressed in the Supplementary material. Equation (16) has up to 6 real roots which, together with the above mentioned relations between components of $\overrightarrow{J}$, specify the stationary values of angular momenta. The stability of a given stationary point can be found from the relation between the radii curvatures of the energy ellipsoid and the angular momentum sphere at the contact point (see the Supplementary material for details).

### Special case: phase transitions in the original LMG

Consider first the special situation with Ω_1_ = Ω_2_ = 0 (or equivalently *K*_1_ = *K*_2_ = 0). This allow us to find the ESQPT of the LMG model studied elsewhere^26–32^ by the new method. To simplify the analysis, assume that *χ*_1,2,3_ > 0 (one can always achieve this by a suitable additive constant), and suppose that *χ*_1,2_ ≠ *χ*_3_. In this case Eq. (16) can be factorized as

17 $$({J}_{3}^{2}-{J}^{2})\,{[{J}_{3}-\frac{{{\rm{\Omega }}}_{3}}{\mathrm{2(}{\chi }_{1}-{\chi }_{3})}]}^{2}\,{[{J}_{3}-\frac{{{\rm{\Omega }}}_{3}}{\mathrm{2(}{\chi }_{2}-{\chi }_{3})}]}^{2}=0.$$

One can see that the stationary angular momenta are always those with *J*_3_ = ±*J* (and thus *J*_1,2_ = 0), and depending on the magnitude of Ω_3_ (or *K*_3_), also the vectors with *J*_3_ = Ω_3_/[2(*χ*_1,2_ − *χ*_3_)]; the existence of the latter cases depends on whether the resulting *J*_3_ fulfills the condition |*J*_3_| < *J*. Thus, the stationary angular momenta are

18 $${\overrightarrow{J}}_{i,ii}=(\begin{array}{c}0\\ 0\\ \pm J\end{array}),\quad {\overrightarrow{J}}_{iii,iv}=(\begin{array}{c}\pm \sqrt{{J}^{2}-{\textstyle \tfrac{{{\rm{\Omega }}}_{3}^{2}}{4({\chi }_{1}-{\chi }_{3}{)}^{2}}}}\\ 0\\ {\textstyle \tfrac{{{\rm{\Omega }}}_{3}}{2({\chi }_{1}-{\chi }_{3})}}\end{array}),\quad {\overrightarrow{J}}_{v,vi}=(\begin{array}{c}0\\ \pm \sqrt{{J}^{2}-{\textstyle \tfrac{{{\rm{\Omega }}}_{3}^{2}}{4({\chi }_{2}-{\chi }_{3}{)}^{2}}}}\\ {\textstyle \tfrac{{{\rm{\Omega }}}_{3}}{2({\chi }_{2}-{\chi }_{3})}}\end{array}).$$

Stationary vectors ${\overrightarrow{J}}_{i,ii}$ occur always, whereas ${\overrightarrow{J}}_{iii,iv}$ occur when |Ω_3_| < 2|*χ*_1_ − *χ*_3_|*J*, and ${\overrightarrow{J}}_{v,vi}$ occur when |Ω_3_| < 2|*χ*_2_ − *χ*_3_|*J*.

Energies of the stationary points are obtained from the Hamiltonian, Eq. (4), by substituting values of ${\overrightarrow{J}}_{i-vi}$ for operators $\hat{\overrightarrow{J}}$. The results correspond to the singular points of the energy spectrum in the limit *N* → ∞. We get

19 $${E}_{i,ii}={\chi }_{3}{J}^{2}\pm {{\rm{\Omega }}}_{3}J,$$

20 $${E}_{iii,iv}={\chi }_{1}{J}^{2}+\frac{{{\rm{\Omega }}}_{3}^{2}}{\mathrm{4(}{\chi }_{1}-{\chi }_{3})},\,\frac{|{{\rm{\Omega }}}_{3}|}{J} < \mathrm{2|}{\chi }_{1}-{\chi }_{3}|,$$

21 $${E}_{v,vi}={\chi }_{2}{J}^{2}+\frac{{{\rm{\Omega }}}_{3}^{2}}{\mathrm{4(}{\chi }_{2}-{\chi }_{3})},\,\frac{|{{\rm{\Omega }}}_{3}|}{J} < \mathrm{2|}{\chi }_{2}-{\chi }_{3}\mathrm{|.}$$

**Figure 4:**
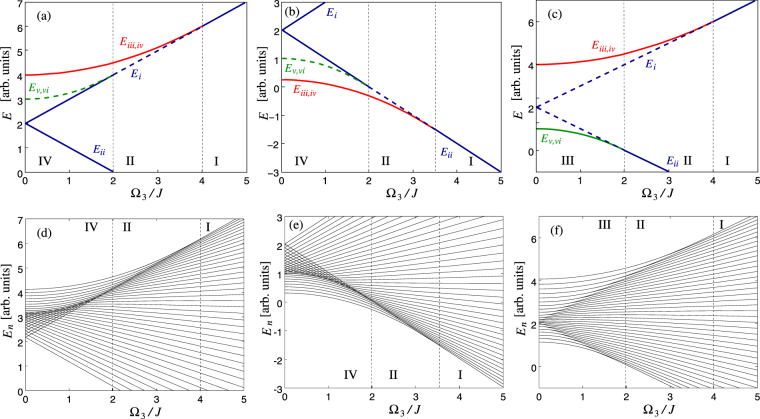
Panels (a–c): Energies of the stationary angular momenta of the original LMG. Full line corresponds to stable, dashed line to unstable values of ${\overrightarrow{J}}_{i-vi}$. Roman numbers I–IV refer to zones specified in ref.^32^. The twisting tensor eigenvalues are (in arbitrary units): (**a**) *χ*_1_ = 4, *χ*_2_ = 3, *χ*_3_ = 2, (**b**) *χ*_1_ = 0.25, *χ*_2_ = 1, *χ*_3_ = 2, (**c**) *χ*_1_ = 1, *χ*_2_ = 4, *χ*_3_ = 2. Panels (d–f): Eigenvalues of the Hamiltonian (4) with Ω_1,2_ = 0 and the values of *χ*_1,2,3_ equal to those of panels (a–c). The number of particles is *N* = 40.

The stability is determined by means of the geomteric considerations in the preceding subsection. The results are shown in Fig. 4(a–c). In Fig. 4(d–f) we show spectra of the corresponding quantum Hamiltonians for comparison: one can see a match between the classical stationary points and the singularities of the quantum spectra (the match is not perfect since a relatively small number *N* was used to make the lines of energy eigenvalues visible).

As a specific example, consider stabilization of rotation of a tennis racket around the middle principal axis by a rotor as in Fig. 1(c). The situation corresponds to Fig. 4(c), and the transition is visualized using the Bloch sphere in Fig. 3. With no rotor (Fig. 3(a)), the sphere consists of two pairs of “self-trapped” regions where motion of the angular momenta encircle the stable directions ±*J*_1_ and ±*J*_2_. These regions are separated by a line called separatrix, going through the unstable stationary angular momenta ±*J*_3_. Adding the rotor with some small angular momentum *K*_3_, the separatrix splits into two (Fig. 3(b)). A new region between the separatrices emerges as a stripe of trajectories encircling the sphere. With increasing |*K*_3_|, the stripe becomes wider and the stable fixed points move towards the unstable points. With a critical value of |*K*_3_|, one pair of stable points merge with one unstable point, resulting in a stable point (Fig. 3(c)). This is a new phase in which the racket co-rotating with the rotor around the intermediate principal axis becomes stable, although counter-rotation is still unstable. With further increasing |*K*_3_|, the remaining pair of stable points approach the unstable point till they merge (Fig. 3(d)). For |*K*_3_| above this second critical value the system is in phase that has only two stationary angular momenta, both stable.

### Phase transitions for general quadratic Hamiltonians

**Figure 5:**
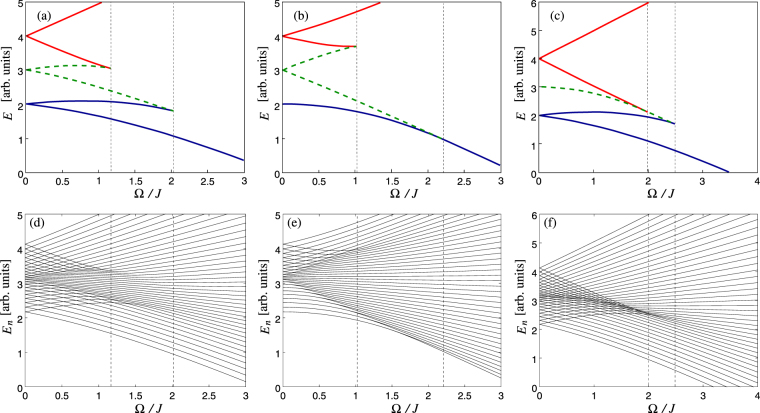
As in Fig. 4, panels (a–c) show energies of the stationary angular momenta and panels (d–f) eigenvalues of the corresponding quantum Hamiltonian (4); in this case, however, $\overrightarrow{{\rm{\Omega }}}$ is not along one of the principal directions of *χ*. The twisting tensor eigenvalues are (in arbitrary units) *χ*_1_ = 4, *χ*_2_ = 3, *χ*_3_ = 2, the ratio of components of vector $\overrightarrow{{\rm{\Omega }}}$ are Ω_1_: Ω_2_: Ω_3_ as follows, (**a**,**d**) 2:1:1, (**b**,**e**) 1:2:0, (**c**,**f**) 2:0:1.

For general values of $\overrightarrow{{\rm{\Omega }}}$ one can factorize Eq. (16) numerically, the results being in Fig. 5(a–c). Panels (d–f) of Fig. 5 show eigenvalues of the corresponding quantum Hamiltonian. The general features are as follows. Starting at $\overrightarrow{{\rm{\Omega }}}=\overrightarrow{0}$, the system has three pairs of degenerate stationary angular momenta with energies *χ*_1,2,3_*J*^2^. Ramping up $|\overrightarrow{{\rm{\Omega }}}|$, the degeneracy is lifted for those stationary angular momenta in whose direction $\overrightarrow{{\rm{\Omega }}}$ has a nonzero component. In Fig. 5(a,d) this is the case for all three components. In Fig. 5(b,c,e,f) one component of $\overrightarrow{{\rm{\Omega }}}$ vanishes and the degeneracy of the corresponding energy remains (note that in the original LMG model in Fig. 4 two components vanish so that only one degeneracy is lifted).

One can see that for a general direction, two critical values of Ω occur: at each of them, one of the local extrema of energy merges with one of the saddle points and these two stationary points disappear. Thus, it is natural to distinguish three generic phases of the generalized LMG system, according to the number of saddle points of energy on the angular momentum sphere: those with zero, one, and two saddle points. In case of various symmetries, more detailed classification may be relevant. In particular, considering the original LMG model in^32^, two zones were identified within the phase with two saddle points: zone III in which the saddles have different energies, and zone IV with energy degenerate saddles and lifted degeneracy of either energy minima or maxima.

Other special cases can be found in the generalized LMG model if $\overrightarrow{{\rm{\Omega }}}$ is confined to a plane perpendicular to one of the principal directions of tensor *χ*. In particular, in the phase with a single saddle point, one of the energy extrema may become degenerate—this is the case of Fig. 5(b,e) (note that in the original LMG, both energy extrema are degenerate in zone II in which a single saddle occurs). In the phase with two saddle points, one of the energy extrema may become degenerate (Fig. 5(b,e)), or the saddle points can be degenerate (Fig. 5(c,f)). These cases can be considered as new sub-phases in the generalized LMG model.

## Floquet time crystals

The concept of time crystals was introduced by F. Wilczek^34^, referring to processes in which spontaneous breaking of time symmetry occurs, in analogy to broken spatial symmetry in usual crystals. Interesting phenomena were predicted for systems with periodic driving as so called “Floquet time crystals” (FTCs)^35^, whose experimental observations have recently been reported^36,37^. In the FTCs, the external driving has period *τ* and thus the Hamiltonian has a discrete time symmetry. Yet, under certain conditions the system behavior breaks this time symmetry and periodic phenomena occur in times corresponding to a multiple of *τ*, i.e., *nτ*. Disorder-induced many-body-localization has been considered to be an important feature of FTCs^35^. Nevertheless, several models of “clean” FTCs in systems without disorder have been recently proposed, among them a FTC in the LMG model^24^. Here the system is initialized in one of the degenerate energy extremal states. Then, a kick rotates the system around the axis of the LMG linear term by *π*. As a result, the system swaps to the other degenerate state. If the kicks occur with period *τ* and the system is initially close to one of the local energy extrema, oscillations of some physical quantities may occur with period 2*τ*. In thermodynamic limit (*N* → ∞), the scheme^24^ is described by a set of classical dynamical equations that in some parameter intervals yield chaotic motion whereas in others regular motion 2*τ* prevails, demonstrating robustness of the phenomenon.

**Figure 6:**
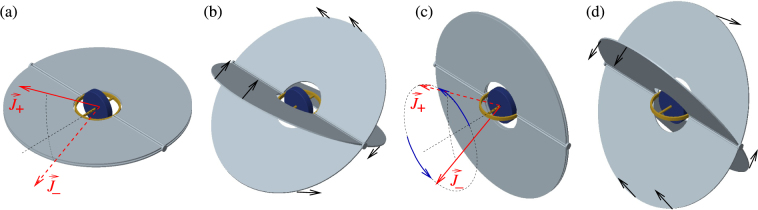
Reshaping a body in the mechanical analogue of the LMG Floquet time crystal. The body starts as a symmetric top with a perpendicular rotor, having two degenerate stable rotational states with angular momenta ${\overrightarrow{J}}_{\pm }$. (**a**) The body is then reshaped (**b**) to take a form of a symmetric top with a coaxial rotor (**c**) so that the original angular momenta ${\overrightarrow{J}}_{\pm }$ precess around the body axis. After swapping ${\overrightarrow{J}}_{\pm }$, the body is reshaped (**d**) back to the original form (**a**).

Our model offers a classical realization of the LMG FTC^24^: assume a plate-like symmetric top with *I*_2_ = *I*_3_ ≡ *I*_0_, and *I*_1_ = 2*I*_0_, with a perpendicular rotor with angular momentum *K*_1,2_ = 0, *K*_3_ ≠ 0 as in Fig. 6(a). Two stable stationary angular momenta occur at ${\overrightarrow{J}}_{\pm }$ with ${J}_{1}=\pm \sqrt{{J}^{2}-4{K}_{3}^{2}}$, *J*_2_ = 0, and *J*_3_ = 2*K*_3_ for any *J* > 2|*K*_3_|. Assume that the system is prepared near one of these stationary points, say ${\overrightarrow{J}}_{+}$ with ${J}_{1}=+\sqrt{{J}^{2}-4{K}_{3}^{2}}$. To swap the stationary states, the body is reshaped, changing its moments of inertia to *I*_1_ = *I*_2_ so as to rotate around *J*_3_; the reshaping happens much faster than the precession. Consider first reshaping to spherical symmetry with *I*_1_ = *I*_2_ = *I*_3_, then according to Eq. (14) the rotational axis precesses with angular velocity $\tilde{{\rm{\Omega }}}={K}_{3}/{I}_{3}$ and after time *τ*_swap_ = *πI*_3_/*K*_3_ the angular momentum swaps to ${J}_{1}=-\sqrt{{J}^{2}-4{K}_{3}^{2}}$. Then, the body reshapes back and continues motion with the rotational axis near the new stationary direction ${\overrightarrow{J}}_{-}$. Assume the body is left to evolve, changing periodically its shape from a symmetric top with perpendicular rotor for time *τ*_0_ to a symmetric top with a coaxial rotor for time *τ*_swap_. The driving period is *τ* = *τ*_0_ + *τ*_swap_, however, the system returns to the initial stationary angular momentum with period 2*τ*, as in a FTC. This behavior is rather stable with respect to variation of system parameters.

**Figure 7:**
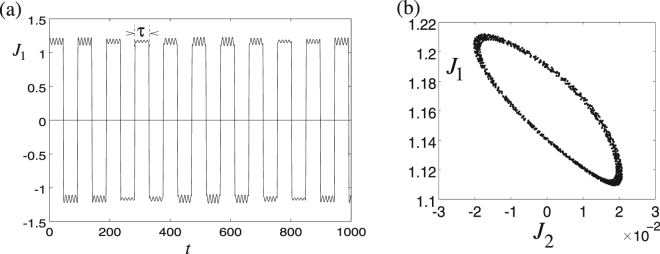
(**a**) Evolution of the angular momentum component *J*_1_ in the mechanical Floquet time crystal scenario. The time and angular momentum are dimensionless, their scales following from the choice *I*_0_ = 1 and *K*_3_ = 1. The initial values are (*J*_1_, *J*_2_, *J*_3_) = (1.2, −0.02, 1.98) and the time parameters are *τ*_0_ = 45.20 and *τ*_switch_ = 2.09. (**b**) Stroboscopic values of *J*_1,2_ for 1000 repetitions.

To study instabilities induced by nonlinear dynamics as in^24^, we consider also reshaping to forms deviating from spherical symmetry, e.g., to *I*_1_ = *I*_2_ = *I*_0_ and *I*_3_ = 2*I*_0_, see Fig. 6(b–d). The rotational axis then precesses with state-dependent angular velocity $\tilde{{\rm{\Omega }}}=({K}_{3}+{J}_{3})/2{I}_{0}$ and the perfect swap after *τ*_swap_ = 2*πI*_0_/(3*K*_3_) is only achieved for states with *J*_3_ = 2*K*_3_. As checked numerically, there are intervals of initial values of *J*_1,2,3_ and of times *τ*_0_ and *τ*_swap_, for which regular motion corresponding to a FTC is observed (see Fig. 7), whereas for other values chaotic behavior occurs.

We can see that LMG model in thermodynamic limit corresponds to a low-degree-of-freedom classical system. This suggests that the transitions between FTC and chaotic behavior in LMG are of similar nature as transitions between regular and chaotic motion in, e.g., driven undamped pendulum, rather than stemming from many-body dynamics.

## Conclusion

Analogies between the rigid bod dynamics and quantum evolution of collective spins allow us to have simple physical pictures of quantum phenomena such as spin squeezing by OAT or by TACT scenarios, or quantum phase transitions in the LMG model. Although thermodynamic limit of the LMG has been widely studied, there has been no classical interpretation proposed so far. Here, by allowing for arbitrary orientation of the rotor axis in the classical domain one can study a generalized LMG model in the quantum domain, predicting new scenarios of quantum phase transitions. These could be observed once a full TACT scheme is implemented (e.g., using the recent proposals^38–40^) with additional suitable linear terms. Vice versa, the LMG Floquet time crystal proposed in the quantum domain^24^ finds its classical counterpart in a periodically reshaped Euler top.

Feynman concludes his wobbling-plate story with enthusiasm^7^: “*I went on to work out equations of wobbles*. *Then I thought about how electron orbits start to move in relativity*. *Then there’s the Dirac Equation in electrodynamics*. *And then quantum electrodynamics*. *[*…*] It was effortless*. *It was easy to play with these things*. *It was like uncorking a bottle*: *Everything flowed out effortlessly*. *I almost tried to resist it*! *There was no importance to what I was doing*, *but ultimately there was*. *The diagrams and the whole business that I got the Nobel Prize for came from that piddling around with the wobbling plate*.” We believe that enthusiasm for physics of wobbling plates is worth sharing and encourage the reader to look for more analogies in the quantum world.

## Electronic supplementary material

Supplemental material
