# Supplementary material for: Analogies of the classical Euler top with a rotor to spin squeezing and quantum phase transitions in a generalized Lipkin-Meshkov-Glick model
Source: Sci Rep. 2018 Jan 31;8:1984. doi: 10.1038/s41598-018-20486-y (PMC5792583; doi:10.1038/s41598-018-20486-y)
Supplement: Supplementary file 1 — Supplemental material [file 41598_2018_20486_MOESM1_ESM.pdf]

Supplemental material for "Analogies of the classical Euler top with a rotor to spin squeezing and quantum phase transitions in a generalized Lipkin-Meshkov-Glick model"

Tomáš Opatrný, Lukáš Richterek, and Martin Opatrný
Faculty of Science, Palacký University, 17. Listopadu 12, 77146 Olomouc, Czech Republic

This document contains supplemental information for the paper *"Euler top with a rotor: classical analogies of spin squeezing and quantum phase transitions in a generalized Lipkin model"* which is referred here to as the "main text". We keep here the abbreviations introduced in the main text, namely LMG for the Lipkin-Meshkov-Glick, OAT for one-axis twisting, and TACT for two-axis countertwisting.

The document is structured as follows. In Sec. I we derive the classical equations of motion. In Sec. II we study the correspondence of energies in the quantum and classical models. Sec. III deals with consequences of transformation of the moments of inertia for the system evolution. In Sec. IV we derive the conditions for stationary angular momenta and their stability in generalized LMG. In Sec. V, ellipsoid principal radii of curvature are derived that are used for finding the stability of the stationary angular momenta. Sec. VI gives an overview of relevant literature.

I. DERIVATION OF THE CLASSICAL EQUATIONS OF MOTION

Evolution of the angular momentum $\vec{L}$ of a rigid body is governed by the equation

$$\frac{d\vec{L}}{dt} = \vec{M}, \tag{I.1}$$

where $\vec{M}$ is the torque. We use the relation between the time derivative $d\vec{A}/dt$ of a vector $\vec{A}$ in an inertial coordinate system and the time derivative $d'\vec{A}/dt$ in a coordinate system that rotates with angular velocity $\vec{\omega}$ with respect to the inertial system

$$\frac{d'\vec{A}}{dt} = \frac{d\vec{A}}{dt} - \vec{\omega} \times \vec{A}. \tag{I.2}$$

Applying that on Eq. (I.1), we get

$$\frac{d'\vec{L}}{dt} = \vec{M} - \vec{\omega} \times \vec{L}. \tag{I.3}$$

Assume that the torque stems from a rotor whose axis is fixed with respect to the rigid body as in Fig. 1 of the main text. We have

$$\vec{M} = -\vec{M}_{\rm rotor} = -\frac{d\vec{K}}{dt}, \tag{I.4}$$

where $\vec{M}_{\rm rotor}$ is the torque with which the rigid body acts on the rotor with angular momentum $\vec{K}$. Using the expression for the time derivative in the rotating system, we have

$$\begin{aligned} \vec{M} &= -\frac{d'\vec{K}}{dt} - \vec{\omega} \times \vec{K} \\ &= -\vec{\omega} \times \vec{K}, \end{aligned} \tag{I.5}$$

since $d'\vec{K}/dt = 0$ as the rotor changes neither the magnitude of rotation nor the axis orientation with respect to the rigid body. Using this in Eq. (I.3) we have

$$\frac{d'\vec{L}}{dt} = -\vec{\omega} \times \left(\vec{L} + \vec{K}\right). \tag{I.6}$$

If the axes of the rotating coordinate system are the principal axes of the tensor of inertia of the body, we have

$$L_k = I_k \omega_k, \qquad k = 1, 2, 3, \tag{I.7}$$

where $I_{1,2,3}$ are the principal moments of inertia. This allows us to write

$$\begin{aligned}
\dot{\omega}_1 &= \frac{I_2 - I_3}{I_1}\omega_2\omega_3 + \frac{K_2\omega_3 - K_3\omega_2}{I_1}, \\
\dot{\omega}_2 &= \frac{I_3 - I_1}{I_2}\omega_3\omega_1 + \frac{K_3\omega_1 - K_1\omega_3}{I_2}, \\
\dot{\omega}_3 &= \frac{I_1 - I_2}{I_3}\omega_1\omega_2 + \frac{K_1\omega_2 - K_2\omega_1}{I_3},
\end{aligned} \tag{I.8}$$

where the dot denotes time derivative in the rotating system. These equations correspond to Eq. (1) of the main text.

Equations (I.8) are the well known Euler dynamical equations which for $\vec{K} = 0$ correspond to a free top, and here the special case corresponds to the torque coming from the rotor. These equations can be expressed in terms of the angular momentum,

$$\begin{aligned}
\dot{L}_1 &= \left(\frac{1}{I_3} - \frac{1}{I_2}\right) L_2L_3 + \frac{K_2}{I_3}L_3 - \frac{K_3}{I_2}L_2, \\
\dot{L}_2 &= \left(\frac{1}{I_1} - \frac{1}{I_3}\right) L_3L_1 + \frac{K_3}{I_1}L_1 - \frac{K_1}{I_3}L_3, \\
\dot{L}_3 &= \left(\frac{1}{I_2} - \frac{1}{I_1}\right) L_1L_2 + \frac{K_1}{I_2}L_2 - \frac{K_2}{I_1}L_1.
\end{aligned} \tag{I.9}$$

It is suitable to work with the total angular momentum $\vec{J} \equiv \vec{L} + \vec{K}$, for which one finds

$$\begin{aligned}
\dot{J}_1 &= \left(\frac{1}{I_3} - \frac{1}{I_2}\right) J_2J_3 + \frac{K_2}{I_2}J_3 - \frac{K_3}{I_3}J_2, \\
\dot{J}_2 &= \left(\frac{1}{I_1} - \frac{1}{I_3}\right) J_3J_1 + \frac{K_3}{I_3}J_1 - \frac{K_1}{I_1}J_3, \\
\dot{J}_3 &= \left(\frac{1}{I_2} - \frac{1}{I_1}\right) J_1J_2 + \frac{K_1}{I_1}J_2 - \frac{K_2}{I_2}J_1.
\end{aligned} \tag{I.10}$$

These equations correspond to Eq. (2) of the main text.

II. CORRESPONDENCE OF ENERGIES IN THE QUANTUM AND CLASSICAL MODELS

The quantum and classical sets of equations correspond to each other provided we make the change described by Eq. (6) of the main text. However, note that whereas there is a straightforward correspondence between the quantum and classical angular momenta $\hat{\vec{J}} \leftrightarrow \vec{J}$, the relation between the energy of the body $E_{\rm body}$ given in Eq. (3) and the quantum Hamiltonian $\hat{H}$ given in Eq. (4) is rather

$$\hat{H} \leftrightarrow -E_{\rm body} + \sum_{k=1}^{3} \frac{K_k^2}{2I_k}. \tag{II.11}$$

The last term is a constant that can be considered trivial. On the other hand, the difference of signs of $\hat{H}$ and $E_{\rm body}$ is interesting: as a result, the quantum vector $\hat{\vec{J}}$ moves on the sphere of $\hat{J}^2 = \text{const}$ along a constant energy contour such that the higher energy area is on the left, the classical vector $\vec{J}$ moves on the sphere of $J^2 = \text{const}$ with the higher energy area on the right.

III. CONSEQUENCES OF TRANSFORMATION OF I_k

As argued in the main text, transformation of I_k and K_k given by Eq. (7) does not change equaions of motion (2). As a consequence, for any quantum system described by twisting tensor χ and frequency vector $\vec{\Omega}$, one can find a classical rigid body characterized by tensor of inertia I supplemented with a rotor with angular momentum $\vec{K}$

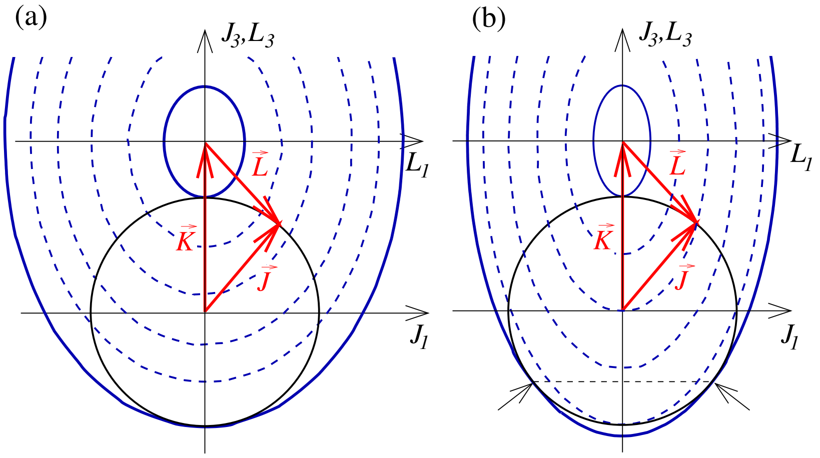

FIG. 1: Constant angular momentum sphere (black) and constant energy ellipsoids (blue) corresponding to a symmetric top with a coaxial rotor. The red vectors $\vec{J}$ and $\vec{L}$ refer to a generic point on the sphere. The maximum and minimum energy ellipsoids compatible with the given value of J are plotted in full line, several other ellipsoids corresponding to intermediate energies are in dashed line. In case (a) with dominant rotation the sphere and each of the extreme energy ellipsoids touch at a single point, in case (b) with dominant nonlinearity the sphere and the maximum energy ellipsoid touch along a circle (indicated by a dashed line and short arrows).

such that the combined system has the same dynamics. To show that, recall that mass can be assembled such as to have arbitrary principal moments of inertia I_k, provided these values are positive and satisfy the triangle inequality $I_j \leq I_k + I_l$ for any permutation of indexes j, k, l. The first condition is satisfied by a suitable choice of the additive constant χ_0 making all values χ_k negative such that all values I_k resulting from Eq. (7) are positive. If the resulting values I_k violate the triangle inequality, one can fix it as follows. Assume that, say, $I_1 > I_2 + I_3$. Then, by choosing I_0 satisfying

$$0 < I_0 < \frac{I_2 I_3 + \sqrt{I_2^2 I_3^2 + I_1 I_2 I_3 (I_1 - I_2 - I_3)}}{I_1 - I_2 - I_3} \tag{III.12}$$

and applying Eq. (7), one finds a realistic tensor of inertia corresponding to the given twisting tensor χ. The linear part of the equations of motion can then easily be adjusted by a suitable choice of $\vec{K}$.

Although the equations of motion are unchanged, note that with J fixed, the energy of the body is then shifted by

$$\Delta E_{\rm body} = \frac{J^2}{2I_0} - \sum_{k=1}^{3} \frac{K_k^2}{2(I_0 + I_k)}. \tag{III.13}$$

Let us also note that the invariance with respect to transformations (6) is valid only for the dynamics of momenta, Eqs. (I.9) or (I.10), but not for the evolution of the angular velocity, Eq. (I.8). This means that one can mutually map the quantum and classical evolution only with respect to where the angular momentum points, but not with respect to how the rigid body is oriented itself. The latter would follow from the kinematic Euler equations which are not included in our study. We anticipate that with expanding the analogy, the classical body orientation would be related to the global phase of the quantum system. Even though connections to other interesting phenomena might be found, such as, e.g., the Montgomery phase [1, 2] referring to the change of body orientation after $\vec{J}$ returns to its initial value, these are beyond the scope of this paper.

IV. STATIONARY ANGULAR MOMENTA AND THEIR STABILITY IN GENERALIZED LMG

A. Stationary values of the angular momentum

In the angular momentum space, stationary values correspond to the points where the constant energy ellipsoid touches the constant total-angular-momentum sphere (see Fig. 1 for an illustration corresponding to a symmetric top with a coaxial rotor). In the classical model, this occurs where the gradient of energy is colinear with the gradient of

the squared total momentum,

$$\text{grad } E_{\text{body}} = \lambda \text{ grad } J^2 \tag{IV.14}$$

for some λ. This leads to the relation between the angular momentum components

$$J_1 = \frac{I_3 K_1 J_3}{(I_3 - I_1)J_3 + I_1 K_3}, \tag{IV.15}$$

$$J_2 = \frac{I_3 K_2 J_3}{(I_3 - I_2)J_3 + I_2 K_3}, \tag{IV.16}$$

which, when used in $J^2 = J_1^2 + J_2^2 + J_3^2$, leads to the polynomial equation for J_3,

$$\sum_{n=0}^{6} a_n J_3^n = 0, \tag{IV.17}$$

where the coefficients a_n are

$$a_0 = -J^2 K_3^4 \frac{I_1^2 I_2^2}{(I_3 - I_1)^2 (I_3 - I_2)^2}, \tag{IV.18}$$

$$a_1 = -2J^2 K_3^3 \frac{I_1 I_2 (I_1 I_3 - 2I_1 I_2 + I_2 I_3)}{(I_3 - I_1)^2 (I_3 - I_2)^2}, \tag{IV.19}$$

$$a_2 = \frac{I_1^2 I_2^2 K_3^4 + I_3^2 K_3^2 \left(I_1^2 K_2^2 + I_2^2 K_1^2\right) - J^2 K_3^2 \left[(I_1 I_3 - 2I_1 I_2 + I_2 I_3)^2 + 2I_1 I_2 (I_3 - I_1)(I_3 - I_2)\right]}{(I_3 - I_1)^2 (I_3 - I_2)^2}, \tag{IV.20}$$

$$a_3 = \frac{2K_3^3 I_1 I_2 (I_1 I_3 - 2I_1 I_2 + I_2 I_3) + 2K_3 I_3^2 \left[I_2 (I_3 - I_2) K_1^2 + I_1 (I_3 - I_1) K_2^2\right]}{(I_3 - I_1)^2 (I_3 - I_2)^2} - \frac{2J^2 K_3 (I_1 I_3 - 2I_1 I_2 + I_2 I_3)}{(I_3 - I_1)(I_3 - I_2)}, \tag{IV.21}$$

$$a_4 = K_3^2 \frac{(I_1 I_3 - 2I_1 I_2 + I_2 I_3)^2 + 2I_1 I_2 (I_3 - I_1)(I_3 - I_2)}{(I_3 - I_1)^2 (I_3 - I_2)^2} + I_3^2 \left[\frac{K_1^2}{(I_3 - I_1)^2} + \frac{K_2^2}{(I_3 - I_2)^2}\right] - J^2 \tag{IV.22}$$

$$a_5 = 2K_3 \frac{I_1 I_3 - 2I_1 I_2 + I_2 I_3}{(I_3 - I_1)(I_3 - I_2)}, \tag{IV.23}$$

$$a_6 = 1. \tag{IV.24}$$

For the quantum mechanical variables the coefficients are

$$a_0 = -\frac{J^2 \Omega_3^4}{16(\chi_1 - \chi_3)^2 (\chi_2 - \chi_3)^2}, \tag{IV.25}$$

$$a_1 = \frac{J^2 \Omega_3^3 (\chi_1 + \chi_2 - 2\chi_3)}{4(\chi_1 - \chi_3)^2 (\chi_2 - \chi_3)^2}, \tag{IV.26}$$

$$a_2 = \Omega_3^2 \frac{\Omega_1^2 + \Omega_2^2 + \Omega_3^2 - 4J^2 \left[(\chi_1 + \chi_2 - 2\chi_3)^2 + 2(\chi_1 - \chi_3)(\chi_2 - \chi_3)\right]}{16(\chi_1 - \chi_3)^2 (\chi_2 - \chi_3)^2}, \tag{IV.27}$$

$$a_3 = \Omega_3 \frac{\Omega_1^2 (\chi_3 - \chi_2) + \Omega_2^2 (\chi_3 - \chi_1) - \Omega_3^2 (\chi_1 + \chi_2 - 2\chi_3)}{4(\chi_1 - \chi_3)^2 (\chi_2 - \chi_3)^2} + J^2 \Omega_3 \frac{\chi_1 + \chi_2 - 2\chi_3}{(\chi_1 - \chi_3)(\chi_2 - \chi_3)}, \tag{IV.28}$$

$$a_4 = \frac{\Omega_3^2 \left[(\chi_1 + \chi_2 - 2\chi_3)^2 + 2(\chi_1 - \chi_3)(\chi_2 - \chi_3)\right]}{4(\chi_1 - \chi_3)^2 (\chi_2 - \chi_3)^2} + \frac{\Omega_1^2}{4(\chi_1 - \chi_3)^2} + \frac{\Omega_2^2}{4(\chi_2 - \chi_3)^2} - J^2, \tag{IV.29}$$

$$a_5 = -\frac{\Omega_3 (\chi_1 + \chi_2 - 2\chi_3)}{(\chi_1 - \chi_3)(\chi_2 - \chi_3)}, \tag{IV.30}$$

$$a_6 = 1. \tag{IV.31}$$

Equation (IV.17) has up to 6 real roots which, together with Eqs. (IV.15) and (IV.16), specify the stationary values of $\vec{J}$.

B. Stationary point stability

There is a simple geometrical picture allowing us to find the stability of a given stationary point. Assume first that the centers of the angular momentum sphere and of the energy ellipsoid are in the same half-space defined by the tangential plane of the contact point. At the point of contact, the energy ellipsoid has two principal radii of curvature, R_1 and R_2. Assume now that both radii are larger than the radius of the sphere, $R_{1,2} > J$. The ellipsoid then touches the sphere from outside. For slightly higher energy there is no contact between the sphere and the ellipsoid, and for slightly lower energy the ellipsoid and the sphere intersect in a closed curve. Thus, the contact point corresponds to a local maximum of energy, i.e., a stable stationary point encircled by states of slightly lower energy. Similarly for both $R_{1,2} < J$ the ellipsoid touches the sphere from inside, and the contact point is stable stationary point of the local energy minimum. On the other hand, if, say, $R_1 < J < R_2$, then there exist two directions along which the ellipsoid radius coincides with J. Along these directions the ellipsoid intersects the sphere. The contact point then corresponds to the energy saddle on the angular momentum sphere, with the intersection lines corresponding to trajectories approaching to or departing from the (unstable) stationary point.

Assume now that the centers of the sphere and of the ellipsoid are in opposite half-spaces defined by the tangential plane of the contact point. Then the sphere and the ellipsoid touch each other from outside and the contact point corresponds to a stable stationary angular momentum.

In Sec. V we derive the principal curvatures at a general point of an ellipsoid. To analyze various phases then means finding stationary points by solving the algebraic equation (IV.17) and deciding about their stability by finding the principal radii of the energy ellipsoid using Eq. (V.64).

C. Special case: phase transitions in the original LMG

Consider first the special situation with $\Omega_1 = \Omega_2 = 0$ (or equivalently $K_1 = K_2 = 0$). To decide about the stability of the stationary angular momenta $\vec{J}_i$–$\vec{J}_{vi}$ of Eq. (18) of the main text, we find the principal curvature radii of the energy ellipsoid at the stationary points as follows,

$$\vec{J}_i: \quad R_1 = \frac{\chi_3}{\chi_1}\left|J + \frac{\Omega_3}{2\chi_3}\right|, \tag{IV.32}$$

$$R_2 = \frac{\chi_3}{\chi_2}\left|J + \frac{\Omega_3}{2\chi_3}\right|, \tag{IV.33}$$

$$\vec{J}_{ii}: \quad R_1 = \frac{\chi_3}{\chi_1}\left|J - \frac{\Omega_3}{2\chi_3}\right|, \tag{IV.34}$$

$$R_2 = \frac{\chi_3}{\chi_2}\left|J - \frac{\Omega_3}{2\chi_3}\right|, \tag{IV.35}$$

$$\vec{J}_{iii,iv}: \quad R_1 = \frac{\chi_1 J}{\chi_3\left(1 - \frac{\Omega_3^2}{4\chi_3(\chi_3-\chi_1)J^2}\right)}, \tag{IV.36}$$

$$R_2 = \frac{\chi_1}{\chi_2}, \tag{IV.37}$$

$$\vec{J}_{v,vi}: \quad R_1 = \frac{\chi_2 J}{\chi_3\left(1 - \frac{\Omega_3^2}{4\chi_3(\chi_3-\chi_2)J^2}\right)}, \tag{IV.38}$$

$$R_2 = \frac{\chi_2}{\chi_1}. \tag{IV.39}$$

Comparing the values $R_{1,2}$ with J according to the criteria in Sec. IV B, we find the following different regimes (see Fig. 4 of the main text).

1. Case $\chi_3 < \chi_2 < \chi_1$ (Fig. 4(a) of the main text): $\vec{J}_i$ is unstable for $2J(\chi_2 - \chi_3) < \Omega_3 < 2J(\chi_1 - \chi_3)$ and stable outside this interval; $\vec{J}_{ii}$ is unstable for $-2J(\chi_1 - \chi_3) < \Omega_3 < -2J(\chi_2 - \chi_3)$ and stable otherwise; $\vec{J}_{iii,iv}$ are stable in the whole interval of their existence $-2J(\chi_1 - \chi_3) < \Omega_3 < 2J(\chi_1 - \chi_3)$ and $\vec{J}_{v,vi}$ are unstable in their whole interval $-2J(\chi_2 - \chi_3) < \Omega_3 < 2J(\chi_2 - \chi_3)$.

   Starting from $\Omega_3 = 0$, the system has two degenerate energy minima, two degenerate saddle points, and two degenerate maxima. Varying Ω_3 in the interval $|\Omega_3| < 2J(\chi_2-\chi_3)$, the degeneracy of the energy minima is lifted,

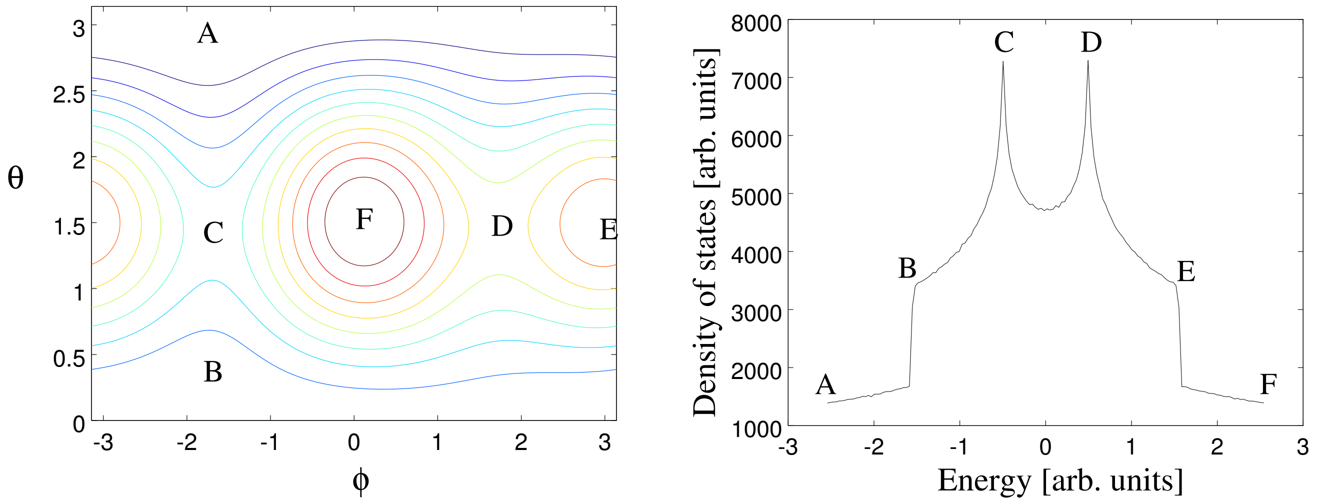

FIG. 2: Left panel: Contours of the equal energy of the generalized LMG model with $(\chi_1, \chi_2, \chi_3) = (2, 0, -2)$ and $(\Omega_1, \Omega_2, \Omega_3) = (0.5, 0.5, 0.5)$. Coordinates ϕ and θ refer to the direction of vector $\vec{J}$. Right panel: energy spectrum corresponding to the same parameters. The beginning and end of the graph correspond to the global minimum and maximum, respectively. The two discontinuities correspond to the local minimum and local maximum, and the peaks correspond to the two saddle points.

and apart from the degenerate saddles and maxima, the system has one global and one local energy minimum; this corresponds to zone IV defined in [3, 4]. At $|\Omega_3| = 2J(\chi_2 - \chi_3)$ the two saddle points merge with the local energy minimum forming a single saddle point, so that in the intervals $2J(\chi_2 - \chi_3) < |\Omega_3| < 2J(\chi_1 - \chi_3)$ the system has one global energy minimum, one saddle point, and two degenerate energy maxima. This interval corresponds to zone II of [3]. At $|\Omega_3| = 2J(\chi_3 - \chi_1)$ the two degenerate maxima and the saddle point merge to form a single global maximum. Then, for $2J(\chi_3 - \chi_1) < |\Omega_3|$ the system has just one global energy minimum and one global maximum, corresponding to zone I of [3].

Note that case $\chi_3 < \chi_1 < \chi_2$ has qualitatively the same properties, except that χ_1 and χ_2 change their roles.

2. Case $\chi_1 < \chi_2 < \chi_3$ (Fig. 4(b) of the main text): the situation is the same as for $\chi_3 < \chi_2 < \chi_1$, except that the terms $\chi_1 - \chi_3$ and $\chi_2 - \chi_3$ change signs, and maxima and minima switch their roles. Similarly for $\chi_2 < \chi_1 < \chi_3$ where χ_1 and χ_2 change their roles.

3. Case $\chi_1 < \chi_3 < \chi_2$ (Fig. 4(c) of the main text): $\vec{J}_i$ is unstable for $-2J(\chi_3 - \chi_1) < \Omega_3 < 2J(\chi_2 - \chi_3)$ and stable outside this interval; $\vec{J}_{ii}$ is unstable for $-2J(\chi_2 - \chi_3) < \Omega_3 < 2J(\chi_3 - \chi_1)$ and stable otherwise. $\vec{J}_{iii-vi}$ are stable in the whole intervals of their existence.

   Assume now, to be specific, that $\chi_3 - \chi_1 < \chi_2 - \chi_3$. Starting from $\Omega_3 = 0$ up to $|\Omega_3| = 2J(\chi_3 - \chi_1)$, the degeneracy of the two saddle points is lifted, while there are still two degenerate maxima and two degenerate minima of energy. This regime corresponds to zone III of [3]. At $|\Omega_3| = 2J(\chi_3 - \chi_1)$ the two minima and the lower-energy saddle point merge to form a single global minimum. In the intervals $2J(\chi_3 - \chi_1) < |\Omega_3| < 2J(\chi_2 - \chi_3)$ the system has one global energy minimum, one saddle point and two global energy maxima, corresponding to zone II of [3]. At $|\Omega_3| = 2J(\chi_2 - \chi_3)$ the two maxima and the saddle point merge to form a single energy maximum; for $|\Omega_3| > 2J(\chi_2 - \chi_3)$ the system has one global energy maximum and one minimum, corresponding to zone I of [3].

D. Phase transitions in the generalized LMG

For general values of $\vec{\Omega}$ one can factorize Eq. (IV.17) numerically. In addition to the results presented in the main text, in Fig. 2 we display energy contours and the density of the energy spectrum for a phase with two nondegenerate unstable stationary angular momenta.

V. PRINCIPAL RADII OF CURVATURE OF AN ELLIPSOID

Consider an ellipsoid

$$\left(\frac{x}{a}\right)^2 + \left(\frac{y}{b}\right)^2 + \left(\frac{z}{c}\right)^2 = 1 \tag{V.40}$$

which can be parametrized as

$$\begin{aligned} x &= a\sin\theta\cos\phi, && \text{(V.41)}\\ y &= b\sin\theta\sin\phi, && \text{(V.42)}\\ z &= c\cos\theta. && \text{(V.43)} \end{aligned}$$

In general, one can calculate the principal curvatures $\kappa_{1,2}$ from the mean curvature $H = (\kappa_1 + \kappa_2)/2$ and Gauss curvature $G = \kappa_1\kappa_2$ as (see, e.g., [5])

$$\kappa_{1,2} = H \pm \sqrt{H^2 - G}, \tag{V.44}$$

where

$$\begin{aligned} H &= \frac{g_{11}h_{22} - 2g_{12}h_{12} + g_{22}h_{11}}{2(g_{11}g_{22} - g_{12}^2)}, && \text{(V.45)}\\ G &= \frac{h_{11}h_{22} - h_{12}^2}{g_{11}g_{22} - g_{12}^2}, && \text{(V.46)} \end{aligned}$$

and

$$\begin{aligned} g_{ij} &= \vec{x}_i \cdot \vec{x}_j, && \text{(V.47)}\\ \vec{x}_i &= \frac{\partial \vec{x}}{\partial u_i}, && \text{(V.48)}\\ h_{ij} &= \vec{n} \cdot \vec{x}_{ij}, && \text{(V.49)}\\ \vec{x}_{ij} &= \frac{\partial^2 \vec{x}}{\partial u_i \partial u_j}, && \text{(V.50)}\\ \vec{x} &= [x, y, z]^T, && \text{(V.51)} \end{aligned}$$

and $\vec{n}$ is a unit normal vector to the surface. For the ellipsoid parametrization we use $u_1 = \phi$ and $u_2 = \theta$. We thus find

$$\vec{x}_1 = \begin{pmatrix} -a\sin\theta\sin\phi \\ b\sin\theta\cos\phi \\ 0 \end{pmatrix}, \qquad \vec{x}_2 = \begin{pmatrix} a\cos\theta\cos\phi \\ b\cos\theta\sin\phi \\ -c\sin\theta \end{pmatrix}, \tag{V.52}$$

so that

$$\begin{aligned} g_{11} &= (a^2\sin^2\phi + b^2\cos^2\phi)\sin^2\theta, && \text{(V.53)}\\ g_{12} &= (b^2 - a^2)\sin\theta\cos\theta\sin\phi\cos\phi, && \text{(V.54)}\\ g_{22} &= (a^2\cos^2\phi + b^2\sin^2\phi)\cos^2\theta + c^2\sin^2\theta. && \text{(V.55)} \end{aligned}$$

The normal vector is found as

$$\vec{n} = \frac{\vec{x}_1 \times \vec{x}_2}{|\vec{x}_1 \times \vec{x}_2|} = -\frac{1}{Q}\begin{pmatrix} bc\sin^2\theta\cos\phi \\ ac\sin^2\theta\sin\phi \\ ab\sin\theta\cos\theta \end{pmatrix}, \tag{V.56}$$

where

$$Q = \left(b^2c^2\sin^4\theta\cos^2\phi + a^2c^2\sin^4\theta\sin^2\phi + a^2b^2\sin^2\theta\cos^2\theta\right)^{1/2}. \tag{V.57}$$

The other important vectors are

$$\vec{x}_{11} = \begin{pmatrix} -a\sin\theta\cos\phi \\ -b\sin\theta\sin\phi \\ 0 \end{pmatrix}, \qquad \vec{x}_{12} = \begin{pmatrix} -a\cos\theta\sin\phi \\ b\cos\theta\cos\phi \\ 0 \end{pmatrix}, \qquad \vec{x}_{22} = \begin{pmatrix} -a\sin\theta\cos\phi \\ -b\sin\theta\sin\phi \\ -c\cos\theta \end{pmatrix}, \tag{V.58}$$

out of which we can calculate

$$h_{11} = \frac{abc\sin^3\theta}{Q}, \tag{V.59}$$

$$h_{12} = 0, \tag{V.60}$$

$$h_{22} = \frac{abc\sin\theta}{Q}. \tag{V.61}$$

We thus find

$$H = \frac{abc}{2}\cdot\frac{a^2(\sin^2\phi+\cos^2\phi\cos^2\theta)+b^2(\cos^2\phi+\sin^2\phi\cos^2\theta)+c^2\sin^2\theta}{(a^2b^2\cos^2\theta+a^2c^2\sin^2\phi\sin^2\theta+b^2c^2\cos^2\phi\sin^2\theta)^{3/2}} \tag{V.62}$$

and

$$G = \frac{a^2b^2c^2}{(a^2b^2\cos^2\theta+a^2c^2\sin^2\phi\sin^2\theta+b^2c^2\cos^2\phi\sin^2\theta)^2} \tag{V.63}$$

which allow us to find $\kappa_{1,2}$ according to (V.44). Expressing then in $\kappa_{1,2}$ angular variables θ,ϕ in terms of the cartesian ones fulfilling Eq. (V.40), and taking the reciprocal value of $\kappa_{1,2}$, one finds the principal radii of the ellipsoid in the form

$$R_{1,2} = \frac{2a^2b^2c^2\left(\frac{x^2}{a^4}+\frac{y^2}{b^4}+\frac{z^2}{c^4}\right)^{3/2}}{a^2+b^2+c^2-x^2-y^2-z^2\pm\sqrt{(a^2+b^2+c^2-x^2-y^2-z^2)^2-4a^2b^2c^2\left(\frac{x^2}{a^4}+\frac{y^2}{b^4}+\frac{z^2}{c^4}\right)}}. \tag{V.64}$$

Note that for a sphere $a=b=c=R$ one finds $R_{1,2}=R$. At the vertex of an ellipsoid, $x=a, y=0, z=0$, Eq. (V.64) yields $R_1=c^2/a$, and $R_2=b^2/a$. Along the equator $z=0$ Eq. (V.64) yields

$$R_1 = \frac{1}{ab}\left(\frac{a^2y^2}{b^2}+\frac{b^2x^2}{a^2}\right)^{3/2}, \qquad R_2 = \frac{c^2}{ab}\left(\frac{a^2y^2}{b^2}+\frac{b^2x^2}{a^2}\right)^{1/2}. \tag{V.65}$$

VI. NOTES ON THE LITERATURE

Since there are many important references that could not be included in the main text, we give here overview of some sources relevant for the studied topics.

A. Classical dynamics

Stabilization of rigid body rotation by rotors was studied in [6–8], using the model of a rigid body with a wheel to describe twisted somersault dynamics in [9, 10], dynamics of the tennis racket in [11–13] and in [14–16] with special focus on the Dzhanibekov effect. Motion of a flipped coin (that we find analogous to the OAT dynamics of spin squeezing) was studied in [17].

B. Quantum dynamics of an asymmetric top

Studies of a quantum mechanical asymmetric top go back to the early days of quantum theory [18–25], however, their goal was finding the Hamiltonian spectrum rather than the squeezing dynamics. Even though recently exact diagonalization of the TACT Hamiltonian was studied [26, 27], there was no discussion about the connection to the quantum asymmetric top. We also note that recently an analogy between the tennis racket motion and a driven two-level system was identified, relevant to spin control in nuclear magnetic resonance [28].

C. LMG model

The model was formulated in 1965 by Lipkin, Meshkov and Glick as a toy model of multiparticle interaction that can be, under certain conditions, solved exactly, and thus serve as a basis for testing various approximation methods [29]. Although the original motivation was in modeling atomic nuclei, the scheme turned out to be useful for studying interesting phenomena in more general systems such as quantum criticality and phase transitions [3, 4, 30–39], multi-particle entanglement [30, 40–43], molecular magnetism [44], or circuit quantum electrodynamics [45].

So far, we are not aware of any experimental realization of a fully controlled, general LMG, although various schemes have been proposed [46–49].

D. Excited state quantum phase transitions

The concept of quantum phase transition typically refers to closing the gap between the ground and the first excited state by varying a system parameter [50]. In contrast to thermal phase transitions where many states are involved and features of the system are suddenly changed by varying temperature, quantum phase transitions can happen at zero temperature. Rather than thermal, the relevant fluctuations are of quantum nature. Recently the concept has been generalized to excited state quantum phase transitions (ESQPT) first studied in [51, 52]. The excited-level dynamics was explored [53], analysis of a single- and two-degrees of freedom systems has been done in [54] and for higher degrees of freedom in [55].

E. Spin squeezing by OAT and TACT

The OAT scenario of spin squeezing was first proposed theoretically by Kitagawa and Ueda [56]. Based on proposals specifying various mechanisms (e.g., [57–61]) it was observed experimentally in hyperfine states of individual atoms [62, 63], in collective spins of atomic samples interacting by spin-dependent collisions [64–67], and by optically mediated dispersive interaction in near-resonant cavities [68]. Other proposals for OAT realization include nuclear spins in quantum dots [47, 69], phonon-induced interactions of spins in diamond nanostructures [70], or cold paramagnetic molecules [71]. OAT with coaxial rotation has been studied in [72] and with perpendicular rotation ("twist-and-turn") in [73].

The TACT process proposed in [56] can yield better squeezing than OAT, however it is much more complicated to be performed with atomic spins than OAT. Therefore, various schemes for achieving effective TACT by applying the OAT Hamiltonian and spin rotations have been proposed [74–78]. Possible physical realizations of TACT were suggested for collective spins based on atomic interactions induced by coherent Raman processes through molecular intermediate states [79, 80], for individual atomic spins by inducing nuclear-electronic spin interaction [63], for Bose-Einstein condensate with spatially modulated nonlinearity [46], for optical fields in resonators with Kerr media [81], nuclear spins via electric quadrupole interaction [47], dipolar spinor condensates [82], or for samples of multilevel atoms interacting with near-resonant cavities [48, 49, 83]. The squeezing procedures can be treated also in multi-mode schemes [84–86].

A simple formula for finding the squeezing rate based on derivatives of a classical Hamiltonian has been given in [87].

F. BEC self trapping

The dynamics due to Hamiltonian $\hat{H} \propto \chi \hat{J}_3^2 + \Omega \hat{J}_1$ was studied in [88] as coherent atomic tunneling between two zero-temperature Bose-Einstein condensates confined in a double-well trap, and in [79] as evolution of a two-component condensate. The linear term $\propto \Omega$ corresponds to tunneling of the atoms between the two wells [88] or to Rabi oscillations between the internal states [79], and the nonlinear term $\propto \chi$ refers to the mutual scattering of the atoms. Circling around a single minimum or maximum energy on the Bloch sphere correspond to the oscillation of the condensate between the two wells, whereas trajectories around one of the two local extrema correspond to *self-trapping* of the condensate in one of the wells. (note that the authors of [88] came up with a classical model equivalent to their equations, namely a *"nonrigid pendulum"*). Another proposed realization of such a Hamiltonian is a Bose-Einstein condensate in a ring trap with an optical lattice [89]: two counterpropagating modes are coupled by a periodic potential that changes the propagation direction of the atoms by Bragg reflection (linear term $\propto \Omega$). Sufficiently strong interaction of the atoms (nonlinear term $\propto \chi$) can keep them self-trapped in one of the rotational modes.

In two-state Bose-Einstein condensates, the limiting case of the linear regime with $|\Omega/\chi| \gg N$ has been been dubbed "Rabi regime" whereas the limiting case of the nonlinear regime with $|\Omega/\chi| \ll 1/N$ the "Fock regime", the transition regime with $1/N \ll |\Omega/\chi| \ll N$ being called "Josephson regime" [90]. As shown in [91], these three regimes correspond to different scaling rules for the dependence of the interferometric phase sensitivity on the atomic number. Experimental observation of transitions between the Josephson and Rabi dynamics in spins of a rubidium Bose-Einstein condensate was reported in [34].

G. Floquet time crystals

The concept of time crystals was introduced by F. Wilczek [92, 93], referring to processes in which spontaneous breaking of time symmetry occurs, in analogy to broken spatial symmetry in usual crystals. Interesting phenomena were predicted for systems with periodic driving as so called "Floquet time crystals" [94–96], whose observations have recently been reported in trapped ions [97] and in nitrogen-vacancy centres in diamond [98]. Recently, Floquet time crystal in the LMG model has been proposed [99]. A detailed review of the time crystals is in [100].

---

[1] R. Montgomery *How much does the rigid body rotate? A Berry's phase from the 18'th century.* Am. J. Phys. **59,** 394 (1991).

[2] J. Natario, *An elementary derivation of the Montgomery phase formula for the Euler top.* J. Geom. Mech. **2,** 113 (2010).

[3] P. Ribeiro, J. Vidal, and R. Mosseri, *Thermodynamical Limit of the Lipkin-Meshkov-Glick Model.* Phys. Rev. Lett. **99,** 050402 (2007).

[4] P. Ribeiro, J. Vidal, and R. Mosseri, *Exact spectrum of the Lipkin-Meshkov-Glick model in the thermodynamic limit and finite-size corrections.* Phys. Rev. E **78,** 021106 (2008).

[5] M. M. Lipschutz, *Schaum's Outline of Differential Geometry.* (McGraw-Hill, New York, 1969).

[6] P. S. Krishnaprasad and C. A. Berenstein, *On the equilibria of rigid spacecraft with rotors.* Systems & Control Letters **4,** 157-163 (1984).

[7] A. M. Bloch, P. S. Krishnaprasad, J. E. Marsden, and G. Sánchez de Alvarez, *Stabilization of rigid body dynamics by internal and external torques.* Automatica **28,** 745-756 (1992).

[8] I. Casu, F. Cret, M. Puta, and A. Voitecovici, *Rigid body with a free spinning rotor and nonlinear stability.* Publicationes Mathematicae - Debrecen **54,** 427-436 (1999).

[9] S. Bharadwaj, N. Duignan, H. R. Dullin, K. Leung, and W. Tong, *The diver with a rotor.* Indagationes Mathematicae **27,** 11471161 (2016).

[10] H. R. Dullin and W. Ton, *Twisting Somersault.* SIAM J. Applied Dynamical Systems **15,** 18061822 (2016).

[11] H. Brody *The moment of inertia of a tennis racket.* Phys. Teach. April, 213-216 (1985).

[12] M. S. Ashbaugh, C. C. Chiconc, and R. H. Cushman, *The Twisting Tennis Racket.* Journal of Dynamics and Differential Equations **3,** 67-85 (1991).

[13] L. Van Damme, P. Mardešić, and D. Sugny, *The tennis racket effect in a three-dimensional rigid body.* Physica D **338,** 17 (2017).

[14] H. Murakami, O. Rios, and T. J. Impelluso, *A Theoretical and Numerical Study of the Dzhanibekov and Tennis Racket Phenomena.* J. Appl. Mech. **83,** 111006 (2016).

[15] O. Rios, T. Ono, H. Murakami, and T. J. Impelluso, *An analytical and geometrical study of the Dzhanibekov and tennis racket phenomena.* Proceedings of the ASME 2016 International Mechanical Engineering Congress and Exposition, IMECE2016 (2016).

[16] A. G. Petrov and S. E. Volodin, *Janibekovs Effect and the Laws of Mechanics.* Doklady Physics **58,** 349 (2013). Original Russian text published in Doklady Akademii Nauk **451,** 399 (2013).

[17] P. Diaconis, S. Holmes, and R. Montgomery, *Dynamical Bias in the Coin Toss.* SIAM Rev. **49,** 211 (2007).

[18] E. E. Witmer, *The quantization of the rotational motion of the polyatomic molecule by the new wave mechanics.* Proc. Nat. Acad. Sci. **13,** 60 (1927).

[19] S. C. Wang, *On the asymmetrical top in quantum mechanics.* Phys. Rev. **34,** 243 (1929).

[20] G. W. King, R. M. Hainer, and P. C. Cross, *The asymmetric rotor I Calculation and symmetry classification of energy levels.* J. Chem. Phys. **11,** 27 (1943).

[21] G. W. King, *The Asymmetric Rotor. VI. Calculation of Higher Energy Levels by Means of the Correspondence Principle.* J. Chem. Phys. **15,** 820 (1947).

[22] C. Van Winter *The asymmetric rotator in quantum mechanics.* Physica **20,** 274 (1954).

[23] I. Lukac and Ya. A. Smorodinskiĭ, *The wave functions of an asymmetric top.* Soviet Physics JETP **30,** 728 (1970).

[24] F. Pan and J. P. Draayer, *Algebraic Solutions for the Asymmetric Rotor.* Annals of Physics **275,** 224 (1999).

[25] V. R. Manfredi and L. Salasnich *Pathological behavior in the spectral statistics of the asymmetric rotor model.* Phys. Rev. E **64,** 066201 (2001).

[26] M. Bhattacharya, *Analytical solvability of the two-axis countertwisting spin squeezing Hamiltonian.* arXiv:1509.08530 [quant-ph] (2015).

[27] F. Pan, Y.-Z. Zhang, and J. P. Draayer *Exact solution of the two-axis countertwisting Hamiltonian.* Annals of Physics **376,** 182 (2017).

[28] L. Van Damme, D. Leiner, P. Mardesic, S. J. Glaser, D. Sugny, *Linking the rotation of a rigid body to the Schrdinger equation: The quantum tennis racket effect and beyond.* Sci. Rep. **7,** 3998 (2017).

[29] H. J. Lipkin, N. Meshkov, A.J. Glick, *Validity of many-body approximation methods for a solvable model: (I). Exact solutions and perturbation theory.* Nuclear Physics **62**, 188 (1965).

[30] J. Vidal, G. Palacios, and R. Mosseri, *Entanglement in a second-order quantum phase transition.* Phys. Rev. A **69**, 022107 (2004).

[31] F. Leyvraz and W. D. Heiss, *Large-N Scaling Behavior of the Lipkin-Meshkov-Glick Model.* Phys. Rev. Lett. **95,** 050402 (2005).

[32] O. Castaños, R. López-Peña, J. G. Hirsch, E. López-Moreno, *Classical and quantum phase transitions in the Lipkin-Meshkov-Glick model.* Phys. Rev. B **74,** 104118 (2006).

[33] H. M. Kwok, W. Q. Ning, S. J. Gu, and H. Q. Lin, *Quantum criticality of the Lipkin-Meshkov-Glick model in terms of fidelity susceptibility.* Phys. Rev. E **78**, 032103 (2008).

[34] T. Zibold, E. Nicklas, C. Gross, and M. K. Oberthaler *Classical Bifurcation at the Transition from Rabi to Josephson Dynamics.* Phys. Rev. Lett. **105,** 204101 (2010).

[35] C. A. Hooley, P. D. Stevenson, *The Lipkin-Meshkov-Glick model: 'quasi-local' quantum criticality in nuclear physics.* arXiv:1102.1583 (2011).

[36] G. Engelhardt, V. M. Bastidas, W. Kopylov, and T. Brandes, *Excited-state quantum phase transitions and periodic dynamics.* Phys. Rev. A **91,** 013631 (2015).

[37] S. Campbell, *Criticality revealed through quench dynamics in the Lipkin-Meshkov-Glick model.* Phys. Rev. B **94,** 184403 (2016).

[38] A. Gallemí, G. Queraltó, M. Guilleumas, R. Mayol, and A. Sanpera, *Quantum Magnetism with Mesoscopic Bose-Einstein Condensates.* Phys. Rev. A **94,** 063626 (2016).

[39] R. Gilmore and D. H. Feng, *Phase transitions in nuclear matter described by pseudospin Hamiltonians.* Nucl. Phys. A **301,** 189 (1978).

[40] S. Dusuel and J. Vidal, *Continuous unitary transformations and finite-size scaling exponents in the Lipkin-Meshkov-Glick model.* Phys. Rev. B **71,** 224420 (2005).

[41] J. Vidal, *Concurrence in collective models.* Phys. Rev. A **73,** 062318 (2006).

[42] R. Orus, S. Dusuel, and J. Vidal, *Equivalence of Critical Scaling Laws for Many-Body Entanglement in the Lipkin-Meshkov-Glick Model.* Phys. Rev. Lett. **101**, 025701 (2008).

[43] J. A. Carrasco, F. Finkel, A. González-López, Miguel A. Rodríguez, and P. Tempesta, *Generalized isotropic Lipkin-MeshkovGlick models: ground state entanglement and quantum entropies.* J. Stat. Mech.-Theory E. **2016,** 033114 (2016).

[44] E. M. Chudnovsky and D. A. Garanin, *Spin tunneling via dislocations in Mn-12 acetate crystals.* Phys. Rev. Lett. **87,** 187203 (2001).

[45] J. Larsen, *Circuit QED scheme for realization of the Lipkin-Meshkov-Glick model.* Europhys. Lett. **90,** 54001 (2010).

[46] T. Opatrný, M. Kolář and K. K. Das, *Spin squeezing by tensor twisting and Lipkin-Meshkov-Glick dynamics in a toroidal Bose-Einstein condensate with spatially modulated nonlinearity.* Phys. Rev. A **91,** 053612 (2015).

[47] Y. A. Korkmaz and C. Bulutay, *Nuclear spin squeezing via electric quadrupole interaction.* Phys. Rev. A **93,** 013812 (2016).

[48] L. Yu, C. Li, J. Fan, G. Chen, T.-C. Zhang, and S. Jia, *Tunable two-axis spin model and spin squeezing in two cavities.* Chin. Phys. B **25,** 050301 (2016).

[49] Y.-C. Zhang, X.-F. Zhou, X. Zhou, G.-C. Guo, and Z.-W. Zhou, *Cavity-assisted single-mode and two-mode spin-squeezed states via phase-locked atom-photon coupling.* Phys. Rev. Lett. **118**, 083604 (2017).

[50] S. Sachdev, *Quantum phase transitions.* Physics World **12,** 33 (1999).

[51] P. Cejnar, M. Macek, S. Heinze, J. Jolie, J. Dobeš, *Monodromy and excited-state quantum phase transitions in integrable systems: collective vibrations of nuclei.* J. Phys. A: Math. Gen. **39,** L515 (2006).

[52] M.A. Caprio, P. Cejnar, and F. Iachello, *Excited state quantum phase transitions in many-body systems.* Annals of Physics **323,** 1106 (2008).

[53] P. Cejnar and P. Stránský, *Impact of quantum phase transitions on excited-level dynamics.* Phys. Rev. E **78,** 031130 (2008).

[54] P. Stránský, M. Macek, P. Cejnar, *Excited-state quantum phase transitions in systems with two degrees of freedom: Level density, level dynamics, thermal properties.* Annals of Physics **345,** 73 (2014).

[55] P. Stránský, P. Cejnar, *Classification of excited-state quantum phase transitions for arbitrary number of degrees of freedom.* Physics Letters A **380,** 2637 (2016).

[56] M. Kitagawa and M. Ueda, *Squeezed spin states.* Phys. Rev. A **47,** 5138 (1993).

[57] D. J. Wineland, J. J. Bollinger, W. M. Itano, and D. J. Heinzen, *Squeezed atomic states and projection noise in spectroscopy.* Phys. Rev. A **50,** 67 (1994).

[58] A. Sørensen, L.-M. Duan, J. I. Cirac, and P. Zoller, *Many-particle entanglement with Bose-Einstein condensates.* Nature **409,** 63 (2001).

[59] A. S. Sørensen and K. Mølmer, *Entangling atoms in bad cavities.* Phys. Rev. A **66,** 022314 (2002).

[60] M. Takeuchi, S. Ichihara, T. Takano, M. Kumakura, T. Yabuzaki, and Y. Takahashi, *Spin squeezing via one-axis twisting*

with coherent light. Phys. Rev. Lett. **94,** 023003 (2005).
[61] M. H. Schleier-Smith, I. D. Leroux, and V. Vuletić, *Squeezing the collective spin of a dilute atomic ensemble by cavity feedback.* Phys. Rev. A **81,** 021804(R) (2010).
[62] S. Chaudhury, S. Merkel, T. Herr, A. Silberfarb, I. H. Deutsch, and P. S. Jessen, *Quantum control of the hyperfine spin of a Cs atom ensemble.* Phys. Rev. Lett. **99,** 163002 (2007).
[63] T. Fernholz, H. Krauter, K. Jensen, J. F. Sherson, A. S. Sørensen, and E. S. Polzik, *Spin squeezing of atomic ensembles via nuclear-electronic spin entanglement.* Phys. Rev. Lett. **101,** 073601 (2008).
[64] C. Orzel, A. K. Tuchman, M. L. Fenselau, M. Yasuda, and M. A. Kasevich, *Squeezed states in a Bose-Einstein condensate.* Science **291,** 2386 (2001).
[65] J. Esteve, C. Gross, A. Weller, S. Giovanazzi, and M. K. Oberthaler, *Squeezing and entanglement in a BoseEinstein condensate.* Nature **455,** 1216 (2008).
[66] C. Gross, T. Zibold, E. Nicklas, J. Esteve, and M. K. Oberthaler, *Nonlinear atom interferometer surpasses classical precision limit.* Nature **464,** 1165 (2010).
[67] M. F. Riedel, P. Böhi, Y. Li, T. W. Hänsch, A. Sinatra, and P. Treutlein, *Atom-chip-based generation of entanglement for quantum metrology.* Nature **464,** 1170 (2010).
[68] I. D. Leroux, M. H. Schleier-Smith, and V. Vuletić, *Implementation of Cavity Squeezing of a Collective Atomic Spin.* Phys. Rev. Lett. **104,** 073602 (2010).
[69] M. S. Rudner, L. M. K. Vandersypen, V. Vuletić, and L. S. Levitov, *Generating entanglement and squeezed states of nuclear spins in quantum dots.* Phys. Rev. Lett. **107,** 206806 (2011).
[70] S. D. Bennett, N. Y. Yao, J. Otterbach, P. Zoller, P. Rabl, and M. D. Lukin, *Phonon-induced spin-spin interactions in diamond nanostructures: application to spin squeezing.* Phys. Rev. Lett. **110,** 156402 (2013).
[71] M. Bhattacharya, *Spin squeezing a cold molecule.* Phys. Rev. A **92,** 063823 (2015).
[72] G.-R. Jin, Y.-C. Liu, W.-M. Liu, *Spin squeezing in a generalized one-axis twisting model.* New J. Phys. **11,** 073049 (2009).
[73] W. Muessel, H. Strobel, D. Linnemann, T. Zibold, B. Juliá-Díaz, and M. K. Oberthaler, *Twist-and-turn spin squeezing in Bose-Einstein condensates.* Phys. Rev. A **92,** 023603 (2015).
[74] Y. C. Liu, Z. F. Xu, G. R. Jin, and L. You, *Spin Squeezing: Transforming One-Axis Twisting into Two-Axis Twisting.* Phys. Rev. Lett. **107**, 013601 (2011).
[75] C. Shen and L. M. Duan, *Efficient spin squeezing with optimized pulse sequences.* Phys. Rev. A **87**, 051801 (2013).
[76] J. Y. Zhang, X. F. Zhou, G. C. Guo, and Z. W. Zhou, *Dynamical Spin Squeezing via Higher Order Trotter-Suzuki Approximation.* Phys. Rev. A **90**, 013604 (2014).
[77] W. Huang, Y.-L. Zhang, C.-L. Zou, X.-B. Zou, and G.-C. Guo, *Two-axis spin squeezing of two-component Bose-Einstein condensates via continuous driving.* Phys. Rev. A **91,** 043642 (2015).
[78] L.-N. Wu, M. K. Tey, and L. You, *Persistent atomic spin squeezing at the Heisenberg limit.* Phys. Rev. A **92,** 063610 (2015).
[79] A. Micheli, D. Jaksch, J. I. Cirac, and P. Zoller, *Many-particle entanglement in two-component Bose-Einstein condensates.* Phys. Rev. A **67,** 013607 (2003).
[80] K. Helmerson and L. You, *Creating massive entanglement of Bose-Einstein condensed atoms.* Phys. Rev. Lett. **87,** 170402 (2001).
[81] T. Opatrný, *Twisting tensor and spin squeezing.* Phys. Rev. A **91,** 053826 (2015).
[82] D. Kajtoch and E. Witkowska, *Spin squeezing in dipolar spinor condensates.* Phys. Rev. A **93,** 023627 (2016).
[83] J. Borregaard, E, D. Davis, G. S. Bentsen, M. H. Schleier-Smith, and A. S. Sørensen, *One- and two-axis squeezing of atomic ensembles in optical cavities.* arXiv:1706.01650 [quant-ph] (2017).
[84] L.-M. Duan, A. Sørensen, J. I. Cirac, and P. Zoller *Squeezing and entanglement of atomic beams.* Phys. Rev. Lett. **85,** 3991 (2000).
[85] I. Kruse, K. Lange, J. Peise, B. Lücke, L. Pezzè, J. Arlt, W. Ertmer, C. Lisdat, L. Santos, A. Smerzi, and C. Klempt, *Improvement of an Atomic Clock using Squeezed Vacuum.* Phys. Rev. Lett. **117,** 143004 (2016).
[86] T. Opatrný, *Quasicontinuous-variable quantum computation with collective spins in multipath interferometers.* Phys. Rev. Lett. **119,** 010502 (2017).
[87] T. Opatrný, *Squeezing with classical Hamiltonians.* Phys. Rev. A **92**, 033801 (2015).
[88] A. Smerzi, S. Fantoni, S. Giovanazzi, and S. R. Shenoy, *Quantum coherent atomic tunneling between two trapped Bose-Einstein condensates.* Phys. Rev. Lett. **79**, 4950 (1997).
[89] M. Kolář, T. Opatrný, and K. K. Das, *Criticality and spin squeezing in the rotational dynamics of a Bose-Einstein condensate on a ring lattice.* Phys. Rev. A **92,** 043630 (2015).
[90] A. J. Leggett, *Bose-Einstein condensation in the alkali gases: Some fundamental concepts.* Rev. Mod. Phys. **73,** 307 (2001).
[91] L. Pezzé, L. A. Collins, A. Smerzi, G. P. Berman, and A. R. Bishop, *Sub-shot-noise phase sensitivity with a Bose-Einstein condensate Mach-Zehnder interferometer.* Phys. Rev. A **72,** 043612 (2005).
[92] F. Wilczek, *Quantum time crystals.* Phys. Rev. Lett. **109,** 160401 (2012).
[93] A. Shapere and F. Wilczek, *Classical time crystals.* Phys. Rev. Lett. **109,** 160402 (2012).
[94] K. Sacha, *Modeling spontaneous breaking of time-translation symmetry.* Phys. Rev. A **91,** 033617 (2015).
[95] D. V. Else, B. Bauer, and C. Nayak, *Floquet time crystals.* Phys. Rev. Lett. **117,** 090402 (2016).
[96] N. Y. Yao, A. C. Potter, I.-D. Potirniche, and A. Vishwanath, *Discrete time crystals: rigidity, criticality, and realizations.* Phys. Rev. Lett. **118,** 030401 (2017).
[97] J. Zhang, P. W. Hess, A. Kyprianidis, P. Becker, A. Lee, J. Smith, G. Pagano, I.-D. Potirniche, A. C. Potter, A.

Vishwanath, N. Y. Yao, and C. Monroe, *Observation of a discrete time crystal.* Nature **543,** 217 (2017).

[98] S. Choi, J. Choi, R. Landig, G. Kucsko, H. Zhou, J. Isoya, F. Jelezko, S. Onoda, H. Sumiya, V. Khemani, C. von Keyserlingk, N. Y. Yao, E. Demler, and M. D. Lukin, *Observation of discrete time-crystalline order in a disordered dipolar many-body system.* Nature **543,** 221 (2017).

[99] A. Russomanno, F. Iemini, M. Dalmonte, and R. Fazio, *Floquet time-crystal in the Lipkin-Meshkov-Glick model.* Phys. Rev. B **95,** 214307 (2017).

[100] K. Sacha and J. Zakrzewski, *Time crystals: a review.* arXiv:1704.03735 (2017).
